# Mapping local variation in household overcrowding across Africa from 2000 to 2018: a modelling study

**DOI:** 10.1016/S2542-5196(22)00149-8

**Published:** 2022-08-03

**Authors:** Michael G Chipeta, Emmanuelle P A Kumaran, Annie J Browne, Bahar H Kashef Hamadani, Georgina Haines-Woodhouse, Benn Sartorius, Robert C Reiner, Christiane Dolecek, Simon I Hay, Catrin E Moore

**Affiliations:** a Big Data Institute, Li Ka Shing Centre for Health Information and Discovery, University of Oxford, Oxford, UK; b Centre for Tropical Medicine and Global Health, University of Oxford, Oxford, UK; c African Institute for Development Policy, Lilongwe, Malawi; d Mahidol–Oxford Tropical Medicine Research Unit, Faculty of Tropical Medicine, Mahidol University, Bangkok, Thailand; e Institute for Health Metrics and Evaluation, University of Washington, Seattle, WA, USA; f Department of Health Metrics Sciences, School of Medicine, University of Washington, Seattle, WA, USA; g Centre for Neonatal and Paediatric Infection, St George's, University of London, London, UK

## Abstract

**Background:**

Household overcrowding is a serious public health threat associated with high morbidity and mortality. Rapid population growth and urbanisation contribute to overcrowding and poor sanitation in low-income and middle- income countries, and are risk factors for the spread of infectious diseases, including COVID-19, and antimicrobial resistance. Many countries do not have adequate surveillance capacity to monitor household overcrowding. Geostatistical models are therefore useful tools for estimating household overcrowding. In this study, we aimed to estimate household overcrowding in Africa between 2000 and 2018 by combining available household survey data, population censuses, and other country-specific household surveys within a geostatistical framework.

**Methods:**

We used data from household surveys and population censuses to generate a Bayesian geostatistical model of household overcrowding in Africa for the 19-year period between 2000 and 2018. Additional sociodemographic and health-related covariates informed the model, which covered 54 African countries.

**Findings:**

We analysed 287 surveys and population censuses, covering 78 695 991 households. Spatial and temporal variability arose in household overcrowding estimates over time. In 2018, the highest overcrowding estimates were observed in the Horn of Africa region (median proportion 62% [IQR 57–63]); the lowest regional median proportion was estimated for the north of Africa region (16% [14–19]). Overall, 474·4 million (95% uncertainty interval [UI] 250·1 million–740·7 million) people were estimated to be living in overcrowded conditions in Africa in 2018, a 62·7% increase from the estimated 291·5 million (180·8 million–417·3 million) people who lived in overcrowded conditions in the year 2000. 48·5% (229·9 million) of people living in overcrowded conditions came from six African countries (Nigeria, Ethiopia, Democratic Republic of the Congo, Sudan, Uganda, and Kenya), with a combined population of 538·3 million people.

**Interpretation:**

This study incorporated survey and population censuses data and used geostatistical modelling to estimate continent-wide overcrowding over a 19-year period. Our analysis identified countries and areas with high numbers of people living in overcrowded conditions, thereby providing a benchmark for policy planning and the implementation of interventions such as in infectious disease control.

**Funding:**

UK Department of Health and Social Care, Wellcome Trust, Bill & Melinda Gates Foundation.

## Introduction

Household overcrowding is associated with higher morbidity and mortality, and with poorer sociodemographic conditions in urban areas (eg, as shown in Beiruit) than in households that are not overcrowded.^1^, ^2^, ^3^, ^4^ Moreover, it is known that transmission of infectious diseases occurs at a higher rate when people are in close contact with others.^5^, ^6^, ^7^, ^8^, ^9^ Although it is difficult to prove that transmission is determined solely by overcrowded conditions, household overcrowding has been implicated in several diseases, such as tuberculosis^10^ and acute respiratory infections, including pneumonia.^11^, ^12^ Infectious diseases that are associated with increased transmission in overcrowded households include SARS-CoV-2, influenza, tuberculosis, meningococcal disease, norovirus, and drug-resistant infections (ie, antimicrobial resistance [AMR]).^8^, ^11^, ^12^, ^13^, ^14^, ^15^, ^16^, ^17^, ^18^

In addition to viral spread, household overcrowding, together with several social factors including poverty and level of education, are associated with an increased carriage risk and increased spread of drug-resistant bacteria in high-income countries and low-income and middle-income countries (LMICs).^13^ Household overcrowding is associated with a higher incidence of tuberculosis^18^, ^19^, ^20^ and a higher carriage risk of resistant *Staphylococcus aureus*.^21^ Urbanisation in LMICs contributes to overcrowding and poor sanitation, which might also be linked to the increase and spread of AMR.^22^, ^23^, ^24^, ^25^

The world population is projected to increase to 9·7 billion people by 2050, with most living in LMICs, primarily in Africa, where countries in Africa comprise eight of the top ten countries that have an annual increase in population of between 2·1% and 3·6%. This population increase—without suitable growth in housing stock—will, in turn, increase overcrowding over time.^26^, ^27^ Housing is a fundamental human right, as defined by the UN, which is essential to human security, nutrition, and health. Housing is also an important social determinant of health; living in poor housing conditions has a profound negative effect on health and wellbeing.^2^, ^3^ WHO defines healthy housing as the physical structure of a dwelling that supports, inter alia, human wellbeing, adequate sanitation, illumination, and sufficient space.^4^, ^28^, ^29^, ^30^ Adequate space is necessary for maintaining clean indoor air, reducing disease transmission risk and noise pollution, and for meeting the occupant's privacy needs.^31^, ^32^ Furthermore, good and adequate housing enables access to basic services, inclusive growth (ie, socioeconomic growth that is distributed fairly across society and creates opportunities for all), and sustainable development.^31^, ^32^ This point is underscored by UN Sustainable Development Goal (SDG) 11, which aims to “ensure access for all to adequate, safe and affordable housing and basic services and upgrade slums by 2030”.^3^

**Research in context**

**Evidence before this study**

We conducted a literature search on household overcrowding in Africa, searching PubMed from January, 2000, to November, 2020. We searched for studies using the terms (“housing” OR “housing conditions” OR “crowding” OR “household overcrowding”) AND (“infectious diseases”) AND (“Africa” OR “low-income” OR “middle-income”) in the title, with no language restrictions. We found several studies published since 2010 that addressed household overcrowding as a risk factor for infectious diseases and antimicrobial resistance, but there were only few studies, and these were for localised settings (countries and sub-national locations). Similarly, we identified several studies addressing the status of housing conditions in specific low-income and middle-income settings. We found no systematic assessments of household overcrowding across the African continent.

**Added value of this study**

In our study, we assessed continent-wide household overcrowding in Africa between the years 2000 and 2018. We estimated the proportions and absolute counts of people living in overcrowded conditions resulting from a rapid growth in population and urbanisation. We used data from countries and islands in Africa from 2000 to 2018 using a geospatial modelling technique that incorporated multiple data sources, including surveys and population censuses. The high resolution maps and data for overcrowding estimates generated in our study could be a useful covariate for predicting several infectious diseases, providing information to inform interventions, including for SARS-CoV-2. To the best of our knowledge, household overcrowding has not been assessed at the continent-wide level over such a long duration. A shortage of information on housing conditions impedes interventions and development planning and implementation, as it prevents a full assessment of the threats presented by household overcrowding in low-income and middle-income countries.

**Implications of all the available evidence**

African countries are experiencing substantial and rapid population and urban growth. These changes can be viewed by policy makers as an opportunity for leveraging public health improvements that also affirm wider human development goals, for example, for effective policies for urban growth that support economic development and the eradication of poverty. The estimates provided in our study could help to direct investments towards the provision of adequate, safe, and affordable housing, and basic services for everyone (Sustainable Development Goal 11).

Many different metrics of household overcrowding have been suggested and applied.^1^, ^33^, ^34^ An overarching definition is any situation in which the number of individuals occupying a dwelling exceeds the capacity of the dwelling space (appendix p 2). This definition can be measured in terms of rooms, bedrooms, or floor area where the shortage of space can result in adverse physical and mental health outcomes.^35^ A standard definition of household crowding, as used by the UN and WHO, is any situation in which more than two people occupy a sleeping space in a dwelling.^4^

When applied with other covariates, household overcrowding patterns can accurately predict and parameterise transmission models of infectious disease.^36^, ^37^, ^38^ Moreover, household overcrowding patterns can inform the design of targeted interventions for infectious diseases and poor living conditions, which are useful for policy making at all levels. However, there is currently a lack of detailed, fine-scale information for the patterns and levels of overcrowding that policy makers can act upon.

Current understanding of overcrowding in households across Africa is based on national data. By combining household survey data, population censuses, and other country-specific household surveys within a geostatistical framework, we aimed to provide household overcrowding estimates at finer spatial resolution (5 × 5 km). Household surveys contain a wealth of information and have previously been used as input data for statistical models (ie, geospatial models) to estimate health outcomes and indicators,^39^, ^40^, ^41^, ^42^, ^43^, ^44^ especially in LMICs, where health registries are either incomplete or non-existent. Our spatiotemporal analysis focuses on household size and the number of sleeping rooms or spaces.

## Methods

### Overview

Geolocated data for the prevalence of household overcrowding from household and census datasets were synthesised by MGC and EPAK, then a two-stage Bayesian model-based geostatistical (MBG) framework was applied to produce fine-scale-resolution (5 × 5 km) estimates of household overcrowding proportions between 2000 and 2018 across Africa. Our analysis covered 54 countries from both mainland Africa and islands for which survey data were available (ie, Cabo Verde, Comoros, Madagascar, and São Tomé & Príncipe). Analytical and model validation steps are described later in this Article and in the appendix (pp 31–32).

### Data

Household overcrowding data were extracted from household surveys by authors MGC and EPAK, including Demographic Health Surveys (DHSs), Multiple Indicator Cluster Surveys, Integrated Public Use Microdata Series population censuses, and other country-specific surveys (appendix p 5). These surveys are regularly conducted in LMICs, use similar formats, and provide internationally comparable data collected over many years. We extracted data for the number of rooms or spaces for sleeping in each household and the number of people who slept in the house the night before the survey for the period between 2000 and 2018. These data were linked to the finest spatial resolution or location available: GPS cluster coordinates (latitudes and longitudes) or the smallest identifiable administrative area level, either level one (which were often states) or administrative level two areas (which were often districts). Administrative districts were resampled to point locations using population-weighted *k*-means clustering^41^ (appendix p 27).

Point data and resampled administrative level data were combined and used as input data for the MBG model. We created a binary outcome indicator, with household overcrowding defined as the ratio of individuals to sleeping rooms higher than two (appendix p 2). In total, we assembled 386 surveys with information on household crowding conditions. Of these, 287 surveys had extractable information covering 78 695 991 households from 54 African countries between 2000 and 2018.

### Analysis and model validation

We fitted a two-stage Bayesian MBG model to estimate the proportion of household overcrowding in any given 5 × 5 km pixel and year to generate predictions for household overcrowding across the African continent at a high resolution. Our chosen methods provided the best out-of-sample predictive performance at the expense of an inferential understanding of the drivers of household overcrowding.

To leverage strength from locations and observations to the entire spatial and temporal domain, we selected socioeconomic and environmental covariates (appendix pp 28–30) and fit a stacked ensemble model to capture possible non-linear effects and complex interactions between covariates (informed by plausibility and importance in the model) and household overcrowding (appendix p 30).^45^ To improve computational stability, account for differences in regional overcrowding patterns, and to allow modelling and assessment of the effects of covariates, the model was fitted separately for each region. For each region, three sub-models were fitted to the dataset using the covariate data as explanatory predictors (appendix pp 28–29): generalised additive models, boosted regression trees, and lasso regression. Each sub-model was fitted using five-fold cross-validation to avoid overfitting, and the out-of-sample predictions from across the five holdouts were compiled into a single comprehensive set of predictions per model. In the second stage, the out-of-sample sub-model predictions were fed into the full geostatistical model as the explanatory covariates when conducting the model fit.

We generated pixel-level 95% uncertainty intervals (UIs) from 1000 draws that were created from the posterior distributions of modelled parameters. We then aggregated pixel-level estimates from the 1000 candidate maps to two sub-national administrative levels (states and districts), as well as national levels. We present results at pixel, district, state, and national level resolutions with the associated UIs. All final model map outputs were shaded grey for each map where the total population density was fewer than ten individuals per 1 × 1 km pixel. The national-level resolution maps provide a between-country comparison of overcrowding, and we assessed the within-country overcrowding variation by calculating the relative deviation in household overcrowding. This estimate was calculated by subtracting the national estimate from each district's estimate and dividing by the national estimate. A national level summary was produced, with comparisons for mean relative deviation for each country between 2000 and 2018 (appendix pp 28–32).

We estimated absolute population counts of people living in overcrowded conditions at pixel, district, state, and national levels on the basis of location-year specific population count raster data extracted from Gridded Population of the World (v4).^46^ The estimated absolute population counts (including UIs) for people living in overcrowded environments were derived by multiplying the proportions of modelled household overcrowding by the population count data for a particular location-year at the draw level, and then by calculating a mean value and 95% CIs. This study complied with the Guidelines for Accurate and Transparent Health Estimates Reporting (appendix pp 38–39).^47^

### Role of the funding source

The funder of the study had no role in study design, data collection, data analysis, data interpretation, or writing of the report.

## Results

**Figure 1:**
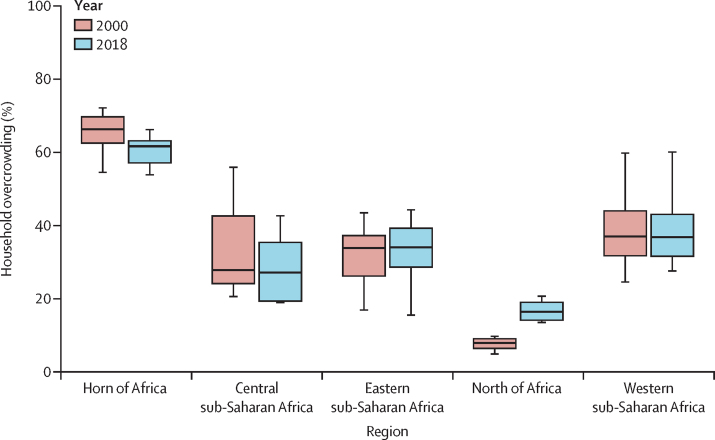
Box and whisker plots of household overcrowding comparisons across Africa by region Median range and IQR of household overcrowding in Africa by modelling regions for the year 2000 (shown in red) and 2018 (shown in blue).

**Figure 2:**
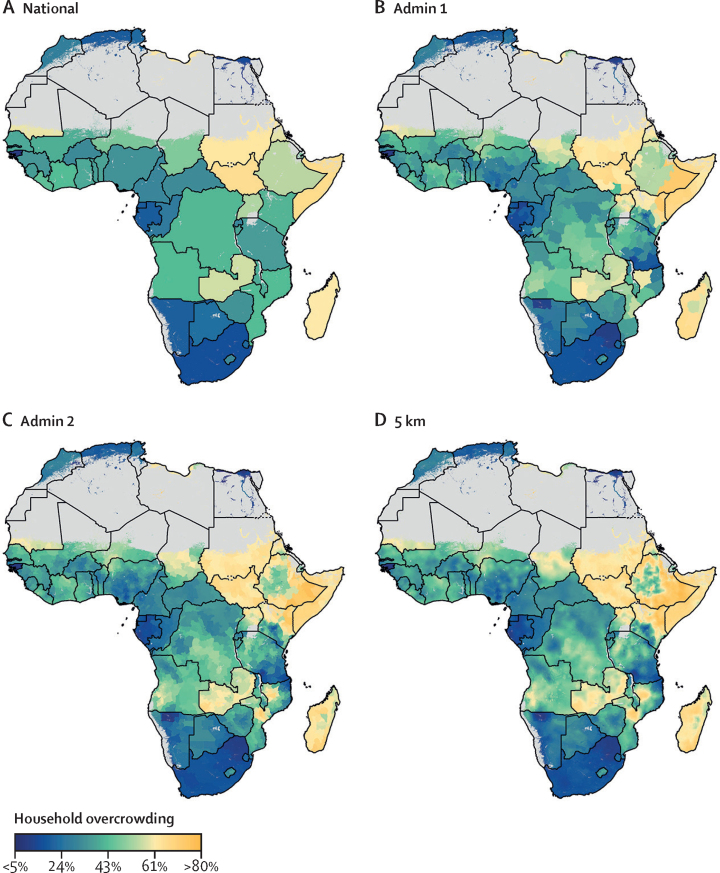
The proportion of overcrowded households in low-income and middle-income countries within Africa, 2018 Modelled estimates are shown by national-level aggregation (A), state (level 1) administrative divisions (B), district (level 2) administrative divisions (C), and 5 × 5 km pixels (D). Pixels (1 × 1 km resolution) with a total population density fewer than ten individuals per 1 × 1 km pixel are shown in grey.

We observed spatiotemporal heterogeneity in household overcrowding estimates, both between and within countries in Africa, between 2000 and 2018. In 2018, the highest prevalence of overcrowding was observed in countries within the Horn of Africa, with a slight decrease observed over the 19-year period (regional median proportion 66% [UI 63–70] in 2000, and 62% [57–63] in 2018). The highest proportions were in Somalia (66% [57–75]), south Sudan (65% [56–74]), and Sudan (63% [53–71]). Although the ratio of overcrowded households in the central sub-Saharan Africa region (out-of-sample *R*^2^ 0·81, appendix pp 35–37) varied widely, it was much lower than in the Horn of Africa region, with a median of 27% [19–35]. The highest proportions were estimated for Zambia (57% [47–66]), Angola (43% [36–49]), and Democratic Republic of the Congo (42% [36–48]). The lowest estimated proportion in central sub-Saharan Africa was for Gabon (19% [14–26%]; Figure 1, Figure 2).

Moderate to high spatial variations were observed in the eastern sub-Saharan Africa region and the southern sub-Saharan Africa region (out-of-sample *R*^2^ 0·58, appendix pp 35–37); the largest differences in household overcrowding in a small spacial area were seen in Madagascar (62%, [54–70]), Kenya (41% [33–49]), and South Africa (15% [12–20]). The eastern sub-Saharan Africa region had a median proportion of 34% (29–39). In the north of Africa region, the highest overcrowding proportion was estimated in Libya at 63% (49–74), while overcrowding was much lower in Egypt (15% [7–27]) and Tunisia (22% [9–42]; figure 2). The regional median proportion doubled in the north of Africa region (out-of-sample *R*^2^ 0·78, appendix pp 35–37; 8% [6–9] in 2000 and 16% [14–19] in 2018; figure 1). The highest overcrowding proportions in the western sub-Saharan Africa region were estimated for Mauritania (60% [45–73]) and Chad (49% [39–59]). The western sub-Saharan Africa region had an estimated median proportion of 37% (32–43; figure 1).

**Figure 3:**
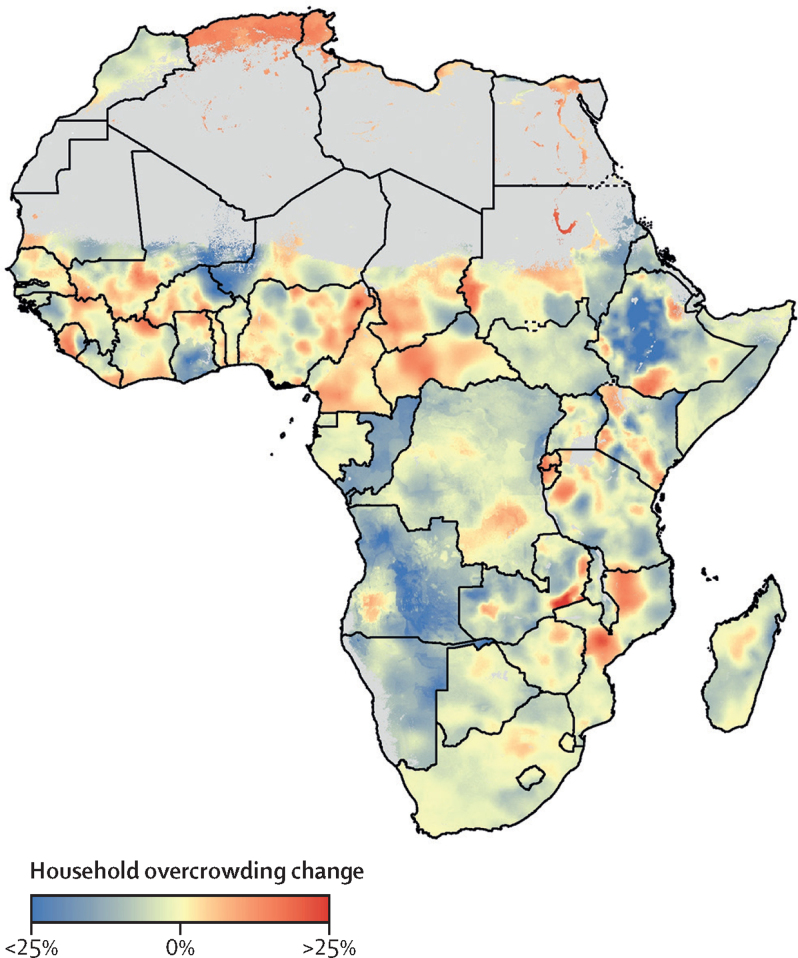
The change in proportion of overcrowded households within Africa from 2000 to 2018 Pixels (1 × 1 km resolution) with a total population density fewer than ten individuals per 1 × 1 km pixel are shown in grey.

Several countries had high overall household overcrowding proportions at the national level in 2018, and the Horn of Africa region consistently had high proportions throughout the study period (Figure 1, Figure 2, Figure 3). A small number of countries had a reduction in overcrowding proportions (eg, Namibia had a reduction of up to 28·8%; table 1). Conversely, some countries had an increase in overcrowding proportions (ranging from 11·4% in Chad to 280% in Algeria; table 2). Most countries in the western, eastern, southern, and central sub-Saharan Africa regions remained relatively unchanged across the study period compared with other studied regions. Sub-nationally, at pixel level (5 × 5 km), household overcrowding had decreased in several countries (figure 3) although overcrowding proportions in these countries remain high.

**Table 1:** Countries in Africa that had the largest decrease (≥10% change) in estimated household overcrowding between 2000 and 2018

	**Household overcrowding estimates, 2000 (%)**	**Household overcrowding estimates, 2018 (%)**	**Percentage change (95% CI)**
**Central sub-Saharan Africa**			
Angola	56%	43%	−23·2% (−46·9 to −21·0)
Namibia	28%	20%	−28·8% (−34·8 to −24·2)
**Western sub-Saharan Africa**			
Niger	54%	48%	−11·1% (−14·0 to −6·3)
Ghana	50%	38%	−24·0% (−28·6 to −19·0)
**Horn of Africa**			
Eritrea	68%	59%	−13·2% (−24·6 to −5·1)
Djibouti	54%	45%	−16·7% (−28·6 to −9·6)
Ethiopia	72%	55%	−23·6% (−27·3 to −19·5)

Modelled estimates at the national level with a relative reduction between 2000 and 2018 and 95% CI of the percentage change.

**Table 2:** Countries in Africa that had the largest increase (≥10% change) in estimated household overcrowding between 2000 and 2018

	**Household overcrowding estimates, 2000 (%)**	**Household overcrowding estimates, 2018 (%)**	**Percentage change (95% CI)**
**North of Africa**			
Algeria	5%	19%	280·0% (200·0–291·0)
Tunisia	11%	22%	100% (50·0–120·0)
**Eastern sub-Saharan Africa**			
Rwanda	19%	30%	57·9% (42·9–63·0)
Burundi	28%	38%	35·7% (28·0–42·1)
**Western sub-Saharan Africa**			
Cameroon	25%	30%	20·0% (15·8–30·0)
Central African Republic	27%	32%	18·5% (10·0–22·0)
Chad	44%	49%	11·4% (8·3–13·0)

Modelled estimates at the national level with a relative increase between 2000 and 2018 and 95% CI of the percentage change.

Within-country variations were also observed. Notably, huge within-country variations (mean relative deviation of districts from the country value) were observed within the central, eastern, and southern sub-Sahara African regions. Namibia (35%) and Mozambique (29%) recorded the highest within-country differences in 2018, followed by Kenya (26%). Egypt (26%) and Nigeria (22%) measured the most significant variations in the north of Africa and western sub-Saharan Africa .

**Figure 4:**
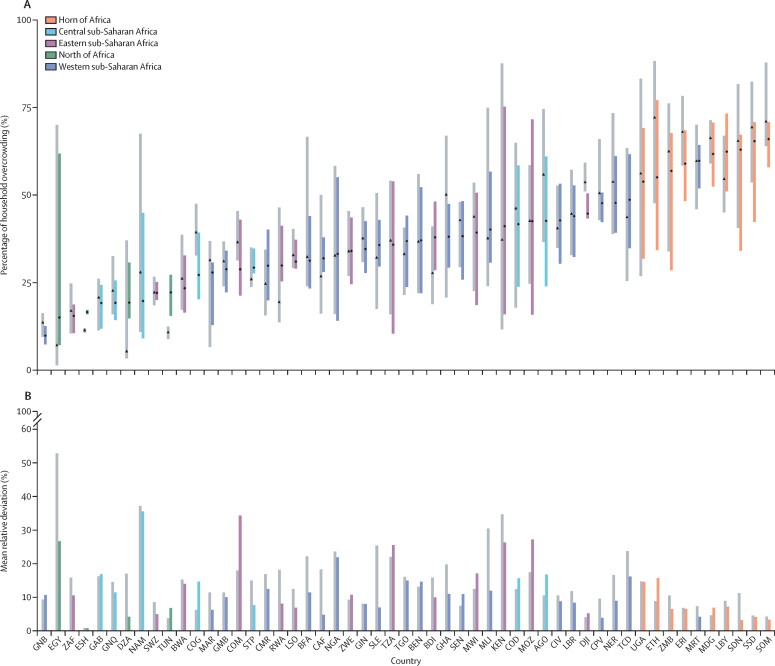
Within-country variation in household overcrowding in 2000 and 2018 (A) Bars show the range in household overcrowding within each country. Grey bars represent estimates for the year 2000, and coloured bars represent estimates for 2018. Black dots represent the mean proportions for household overcrowding for each country. (B) Bars show the mean relative deviation of household overcrowding in administrative level 2 (districts) from the national level household overcrowding estimates. Grey bars represent estimates for the year 2000, and coloured bars represent estimates for 2018. The 2018 colours are based on the country's region, and countries are ordered (along the x-axis) on the basis of mean overcrowding proportions in the year 2018 (low to high). Countries are labelled using International Organization for Standardization (ISO) codes. AGO=Angola. BEN=Benin. BDI=Burundi. BFA=Burkina Faso. BWA=Botswana. CAF=Central African Republic. CIV=Cote d'Ivore. CMR=Cameroon. COD=Congo (the Demographic Republic of the). COG=Congo. COM=Comoros. CPV=Cabo Verde. DJI=Djibouti. DZA=Algeria. EGY=Egypt. ERI=Eritrea. ESH=Western Sahara. ETH=Ethiopia. GAB=Gabon. GHA=Ghana. GIN=Guinea. GMB=Gambia. GNB=Guinea Bissau. GNQ=Equatorial Guinea. KEN=Kenya. LBR=Liberia. LBY=Libya. LSO=Lesotho. MAR=Morocco. MDG=Madagascar. MLI=Mali. MOZ=Mozambique. MRT=Mauritania. MWI=Malawi. NAM=Namibia. NER=Niger. NGA=Nigeria. RWA=Rwanda. STP=Sao Tome and Principe. TGO=Togo. SDN=Sudan. SEN=Senegal. SLE=Sierra Leone. SOM=Somalia. SSD=South Sudan. SWZ=Eswatini. TCD=Chad. TUN=Tunisia. TZA=Tanzania, the United Republic of. UGA=Uganda. ZAF=South Africa. ZMB=Zambia. ZWE=Zimbabwe.

The countries that exhibited the least relative deviations from the national mean in 2018 included Sudan, Somalia, and Cabo Verde (all between 3% and 4%; figure 4). In Egypt, the mean relative deviation of household overcrowding decreased the most between 2000 and 2018, from 53% to 26%. Significant reductions were evident in Sierra Leone (25% to 7%), Central African Republic (18% to 4%), Algeria (17% to 4%), and Rwanda (18% to 8%). Household overcrowding inequality increased over the study period in many countries in sub-Saharan Africa; as shown by the rising mean relative deviation in Comoros (18% to 34%), Republic of the Congo (6% to 14%), Senegal (7% to 11%), and Malawi (12% to 16%; figure 4).

**Figure 5:**
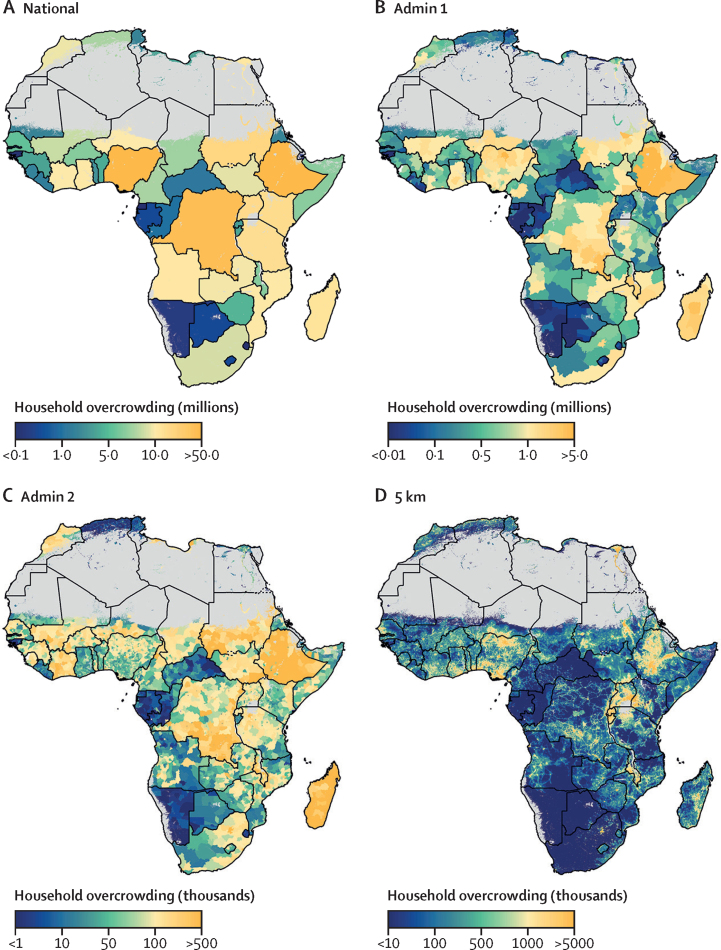
Population counts of people living in overcrowded conditions in Africa, 2018 Modelled estimates are shown by national level aggregation (A), state (level 1) administrative divisions (B), district (level 2) administrative divisions (C), and 5 × 5 km pixels (D). Pixels (1 × 1 km resolution) with a total population density fewer than ten individuals per 1 × 1 km pixel are shown in grey.

The absolute population counts living in overcrowded conditions increased over the 19-year study period, along with population increases for Africa in general. In 2018, a total of 474·4 million (95% UI 250·1–740·7 million) people lived in overcrowded conditions in Africa; an increase from 291·5 million (180·8 million–417·3 million) in 2000. 14 countries accounted for approximately 70% of the 2018 total population living in overcrowded conditions (Nigeria, Ethiopia, Democratic Republic of the Congo, Sudan, Uganda, Kenya, Tanzania, Madagascar, Egypt, Angola, Ghana, Mozambique, Cote d’Ivoire, and Niger; figure 5). Of these, more than 20 million people lived in overcrowded housing conditions in each of the following six countries in sub-Saharan Africa in 2018: Nigeria, Ethiopia, Democratic Republic of the Congo, Sudan, Uganda, and Kenya (table 3). These six countries have a combined population of 538·3 million, which account for 48·5% of the total population (229·9 million people) who are living in overcrowded conditions in Africa.

**Table 3:** Absolute population counts and proportions of people living in overcrowded conditions in 2018

	**Absolute counts**			**Proportions (%, UI)**
	Population in 2018	Population living in crowded conditions in urban areas	Population living in crowded conditions	
Nigeria	202 647 467	33 964 499	65 316 345 (33 184 819–104 960 938)	32·2% (16·4–51·8)
Ethiopia	98 423 665	11 264 486	53 640 411 (32 469 598–73 608 502)	54·5% (33·0–74·8)
Democratic Republic of the Congo	106 226 798	20 304 631	44 140 503 (24 744 843–65 560 635)	41·6% (23·3–61·7)
Sudan	40 415 598	8 884 130	25 383 229 (14 378 490–34 298 159)	62·8% (35·6–84·9)
Uganda	39 362 904	5 473 563	21 052 164 (11 665 350–29 875 815)	53·5% (29·6–75·9)
Kenya	51 227 095	5 720 113	20 428 974 (12 160 791–29 547 393)	39·9% (23·7–57·7)
United Republic of Tanzania	52 806 810	6 739 765	18 215 581 (10 659 356–26 990 652)	34·5% (20·2–51·1)
Madagascar	25 374 824	6 013 238	15 418 558 (9 654 250–20 385 703)	60·8% (38·1–80·3)
Egypt	90 886 136	5 722 960	13 309 209 (2 530 504–36 212 272)	14·6% (2·8–39·8)
Angola	30 278 009	8 540 180	12 746 537 (7 501 606–18 430 408)	42·1% (24·8–60·9)
Ghana	30 660 398	6 635 358	11 640 979 (6 253 018–17 850 364)	38·0% (20·4–58·2)
Mozambique	28 208 179	4 388 076	11 547 569 (7 308 287–16 114 246)	41·0% (25·9–57·1)
Cote d'Ivoire	24 927 552	5 513 889	10 811 548 (5 164 149–17 047 356)	43·4% (20·7–68·4)
Niger	21 557 506	1 752 480	10 308 704 (5 267 144–15 526 592)	47·8% (24·4–72·0)
Zambia	17 295 807	4 413 724	9 808 276 (5 386 556–13 791 360)	56·7% (31·1–79·7)
Morocco	34 451 860	6 073 526	9 489 884 (3 370 343–18 238 191)	27·6% (9·8–52·9)
South Sudan	14 388 976	2 349 261	9 397 044 (5 587 479–12 377 392)	65·3% (38·8–86·0)
South Africa	57 664 805	6 033 202	9 004 779 (4 166 378–16 148 805)	15·6% (7·2–28·0)
Mali	21 153 774	3 749 992	8 522 710 (4 053 278–13 631 197)	40·3% (19·2–64·4)
Algeria	41 298 619	5 759 785	7 890 116 (1 059 259–22 990 741)	19·1% (2·6–55·7)
Cameroon	26 217 798	4 384 206	7 828 940 (3 222 513–14 086 387)	29·9% (12·3–53·7)
Chad	15 236 208	1 706 482	7 419 488 (3 894 663–10 988 639)	48·7% (25·6–72·1)
Somalia	10 302 581	3 193 124	6 793 880 (4 082 442–8 902 759)	66·0% (39·6–86·4)
Malawi	17 278 582	1 214 981	6 749 896 (4 213 903–9 557 598)	39·1% (24·4–55·3)
Burkina Faso	21 124 265	2 032 739	6 557 222 (3 440 139–10 426 965)	31·0% (16·3–49·4)
Senegal	14 315 438	2 648 738	5 405 588 (2 650 556–8 616 317)	37·8% (18·5–60·2)
Zimbabwe	14 001 902	1 800 123	4 737 167 (2 492 124–7 467 873)	33·8% (17·8–53·3)
Benin	11 598 872	2 072 793	4 318 319 (2 103 366–6 938 784)	37·2% (18·1–59·8)
Libya	6 489 464	3 132 790	4 016 397 (2 241 637–5 443 987)	61·9% (34·5–83·9)
Burundi	10 645 769	561 779	4 012 704 (2 207 582–6 072 299)	37·7% (20·7–57·0)
Guinea	11 225 360	1 516 844	3 889 343 (1 575 704–6 852 329)	34·7% (14·0–61·0)
Rwanda	12 744 898	684 120	3 800 669 (2 014 509–6 054 304)	29·8% (15·8–47·5)
Togo	7 813 533	1 259 639	2 929 392 (1 556 752–4 527 726)	37·5% (19·9–58·0)
Tunisia	10 971 254	1 690 079	2 414 398 (400 031–6 366 743)	22·0% (3·7–58·0)
Eritrea	4 046 296	1 503 944	2 387 213 (1 337 935–3 309 249)	59·0% (33·1–81·8)
Mauritania	4 062 871	1 304 511	2 288 615 (1 244 084–3 179 340)	56·3% (30·6–78·3)
Sierra Leone	6 330 537	947 786	2 204 153 (964 819–3 730 522)	34·8% (15·2–58·9)
Liberia	4 174 870	965 649	1 821 979 (940 839–2 768 940)	43·6% (22·5–66·3)
Central African Republic	5 088 636	701 933	1 632 402 (639 560–2 948 472)	32·1% (12·6–57·9)
Congo	3 654 201	695 841	994 059 (494 755–1 650 515)	27·2% (13·5–45·2)
Lesotho	1 831 475	175 015	564 565 (287 490–919 886)	30·8% (15·7–50·2)
Gambia	2 000 268	325 558	551 794 (221 268–999 225)	27·6% (11·1–50·0)
Botswana	2 302 885	387 356	530 625 (232 917–951 986)	23·0% (10·1–41·3)
Gabon	2 640 378	439 256	504 892 (225 794–914 299)	19·1% (8·6–34·6)
Namibia	2 353 714	258 068	469 214 (224 395–814 837)	19·9% (9·5–34·6)
Djibouti	1 046 353	350 906	444 185 (212 648–698 895)	42·5% (20·3–66·8)
Swaziland	1 077 903	71 357	237 855 (110 093–421 245)	22·1% (10·2–39·1)
Equatorial Guinea	1 160 002	164 146	224 857 (93 731–422 955)	19·4% (8·1–36·5)
Cape Verde	475 345	150 916	221 935 (90 719–358 781)	46·7% (19·1–75·5)
Comoros	716 948	53 039	182 892 (81 141–316 636)	25·5% (11·3–44·2)
Guinea-Bissau	1 628 720	70 486	156 635 (48 717–356 027)	9·6% (2·9–21·9)
Sao Tome and Principe	174 326	37 754	51 019 (25 581–83 653)	29·3% (14·7–47·9)
Western Sahara	448 814	5	12 (5–21)	0

Absolute population counts and proportions at the national level, with 95% UI in absolute terms. UI=uncertainty interval.

The five sub-national locations with the highest number of people living in overcrowded conditions in 2018 were: north and south Nigeria; southwest Democratic Republic of the Congo; north, southeast, and central Ethiopia; north Madagascar; east Sudan; and northeast Kenya. Pixel-level data (5 × 5 km) from 2018 shows that the north and south Nigeria, central and north Ethiopia, central and south Uganda, central and southern parts of Madagascar, and central and southern parts of Malawi had the highest population of overcrowded people (figure 5).

## Discussion

The objectives of this modelling study were to quantify the changes in household overcrowding across Africa to a fine spatial resolution over a 19-year period (2000 to 2018) using available country-specific household survey and census data. Given the housing crisis and population growth in Africa, together with the impacts of climate change (eg, droughts and flooding) and the rising spread of infectious diseases, we wanted to examine levels of household overcrowding and assess whether these data should become part of policy for local governments going forward. In our analysis, we found that household overcrowding was stable or decreasing over time in most regions in Africa, but that there was an increase in household overcrowding in north Africa based on the results in the included surveys. Although these surveys can differ between countries, the same questions are repeated over many years, indicating that these data are robust. High proportions of household overcrowding were localised in urban areas, with heterogeneity observed across and within countries. Our findings highlight important spatiotemporal disparities in household overcrowding and provided estimates and UIs for locations where data were sparse.

Housing is explicitly outlined in target 11 of the UN SDGs^3^, ^48^ and is implicitly an important component of other SDGs. Policy makers view reductions in household overcrowding as a key prerequisite for inclusive growth, alongside efforts to reduce poverty, unemployment, and inequality. Estimates of household overcrowding modelled at 5 × 5 km resolution have potentially far-reaching applications in strategies and policies intended to ensure the achievement of the SDGs. Our study highlights countries and areas that could be targeted with interventions (eg, household interventions or specific water, sanitation, and hygiene interventions) to combat infectious disease, including the ongoing SARS-CoV-2 pandemic across Africa.

In our analysis, a marked increase in household overcrowding proportions was observed in north Africa, particularly in Algeria, which had a 280% increase over the 19-year study period. One of the reasons for the increase might be increasing urbanisation, with 78% of the population living in urban areas,^49^ together with a lack of planning and rapid city growth, which has led to extremely high housing deficits. Between 2000 and 2015, the annual urbanisation growth rate average was 2·8% in Algeria, with a housing deficit backlog of 1·2 million households.^50^

Nine of the 54 countries analysed in our study had considerable reductions in household overcrowding proportions (between 9% and 29%). The largest decrease in household overcrowding proportions was observed in Ethiopia (table 1). Until 2005, Ethiopia was one of the least urbanised countries on the African continent. This position has since changed rapidly. Ethiopia now has one of the fastest growing economies, with an average annual growth of 10·5% (measured between 2004 and 2018). In 2018, the urban population of Ethiopia was estimated to account for 21·2% of its 112 million people, and its urbanisation rate stood at 4·9%. From 2006 onward, the Ethiopian Government invested heavily in social housing projects, attracting foreign and local building contractors, promoting integration, and reducing household overcrowding. Ethiopia's Government integrated housing development programme condominium scheme is the most successful local housing programme;^51^ at least 400 000 condominiums have been built against a target of 580 000 units over a 5 year period.^51^, ^52^ Despite this progress, a backlog of approximately 1·2 million housing units exists, with a projected demand of 655 800 housing units needed between 2015 and 2025.^51^

Other exemplar countries include Angola, Namibia, and Zambia. In Angola, the government has established the housing promotion fund (HPF) to promote access to affordable housing. The HPF is responsible for 70% of state-built housing, and the government has encouraged private developers to contribute to the national housing market for mass-scale housing production.^51^ A deficit of 1·7 million housing units exists in Angola. Similarly, although Zambian and Namibian national governments have invested considerably in housing, the housing deficits remain high. Overall, there is a deficit of 68·8 million housing units for people living in overcrowded conditions in Africa.

Improvements in housing conditions in Africa were observed in dwellings (based on sufficient living areas, improved water and sanitation, and durable construction), rising from 11% in 2000 to 23% in 2015.^53^ However, high levels of household overcrowding persist, with Nigeria, Ethiopia, Democratic Republic of the Congo, Sudan, Uganda, Kenya, and Tanzania showing the highest absolute population living in overcrowded conditions in 2018. No material changes in household overcrowding proportions were observed throughout eastern, western, and central sub-Saharan Africa over the 19-year study period; these areas remain overcrowded (above a median of 27%).

Africa is the world's least urbanised continent, and has 11·3% of the world's urban population. The sub-Saharan African region is the least urbanised region; however, the region's cities are rapidly expanding.^54^ The African population has been growing, on average, 2·5% per year between 2000 and 2018. Along with this population growth and urbanisation, the absolute population living in overcrowded conditions has increased drastically, with small increases in housing. Additionally, most migrants from rural areas are uneducated or unskilled and end up in the informal sector in Africa, accounting for 93% of all new jobs and 61% of all urban employment.^55^ Because of the low and intermittent incomes generated by the informal sector, most migrants become slum dwellers or seek housing with slum landlords. Many governments in Africa have directly provided housing to meet the needs of growing urban populations,^51^, ^56^ but these programmes are expensive, difficult for the urban poor to afford, and have yet to result in considerable increases in affordable housing. As a result, the population living in overcrowded conditions increased by 62·7%, with most of these populations living in urban slums.

Urban growth rates are high on the African continent. Projections predict that Africa's cities will be home to an additional 950 million people by 2050.^50^ Most of this growth will likely occur in small-sized and medium-sized towns and cities, which often struggle to attract the required infrastructure investment.^49^ Without substantial investment in housing facilities and services, overcrowding and slum proliferation could increase exponentially around cities. Without essential infrastructure, large populations live in unhealthy, polluted households in congested and poor conditions with poor sanitation, insecure living conditions, and little access to utilities such as electricity and water (Frostad J J, unpublished).

Overcrowded conditions provide the perfect environment for the transmission of epidemics such as SARS-CoV-2 and other infectious diseases. Household overcrowding is positively correlated with the spread of SARS-CoV-2.^5^, ^8^ Globally, governments have implemented non-pharmaceutical interventions to reduce the spread of SARS-CoV-2, which have included partial or total lockdowns of entire regions or countries. These measures have meant that people spend more time at home, many of whom, especially in Africa, are in overcrowded conditions.^57^, ^58^ Living in overcrowded housing conditions makes it harder to self-isolate and shield from SARS-CoV-2. There is a correlation between transmissibility and infection outcomes with SARS-CoV-2 for those living in overcrowded spaces.^5^, ^8^, ^59^, ^60^, ^61^, ^62^ In England, it is estimated that there were at least 70% more cases of SARS-CoV-2 in households that were overcrowded compared with non-overcrowded households.^7^ The unavoidable proximity and use of shared facilities that are inevitable with overcrowding are ideal for the spread of an airborne microbe and can increase the speed and spread of pandemics.

Our model is useful for understanding spatiotemporal differences in household overcrowding, and could also be useful for examining AMR and the spread of SARS-CoV-2 in order to shape policy across Africa. Our high-resolution overcrowding estimates could be used to improve forecasting of infectious diseases, potentially providing valuable information to assist with disease control interventions. For example, our method could be used immediately to model household overcrowding with SARS-CoV-2 and other infectious diseases, such as *Mycobacterium tuberculosis*, as well as AMR, in addition to other research in the area.^13^, ^18^, ^23^, ^59^, ^63^, ^64^, ^65^, ^66^ In a systematic review,^67^ overcrowded conditions were a strong enabler of AMR in Chilean hospitals between 2008 and 2017. Our model, therefore, can be used to determine areas of high overcrowding and high transmission, and help to target interventions to reduce the transmission of infectious diseases.^68^

The main limitations of our study were that we provide estimates in locations without empirical data, and that our data were derived from census data and other country-specific surveys. While these are currently the only sources of representative and comparable data, they contain multiple potential biases, such as recall and reporting bias, interviewer effects on responses, and refusal bias.^69^, ^70^, ^71^, ^72^ In addition, there were data gaps in some locations (appendix p 4) that affected the robustness of our estimates, and increased uncertainty. Our resampling method increased the uncertainty intervals in our analysis. Given that our data maximised predictive performance without providing evidence of causality, additional groundwork is needed to provide this information. There are also possibly new covariates that could have an effect on our outcomes. The ideal next steps would be to conduct additional work to examine the truth of the DHS surveys in small areas, which would enhance the data already available, as well as to conduct new surveys where no data currently exists.

Additionally, our analysis used proxy information to infer urbanicity. As such, further analyses that delineate urban and rural splits would be ideal. There is an urgent need to establish and promote household surveillance programmes to accurately estimate overcrowding conditions across Africa. Household overcrowding definitions need to be harmonised (appendix p 2). We applied the definition of overcrowding as having more than two people per sleeping room. Household overcrowding is a global concern, especially in countries with humanitarian crises. The modelling of household overcrowding needs to be expanded beyond Africa. Despite these limitations, to the best of our knowledge, our study provides the first Africa-wide estimates for household overcrowding using geospatial tools, and highlights the need for interventions to increase housing infrastructure across Africa.

Our data show that 38% of the 1·26 billion people in Africa live in overcrowded households. We also provide the first detailed baseline metrics for overcrowding that build on existing measurements of housing in Africa. These results were limited to urban areas that are not currently standardised at the sub-national scale and were derived from simplistic extrapolations from survey data. Using survey data, we also provide estimates of household overcrowding at fine spatial resolutions across the entire African continent. We found that the number of people living in overcrowded conditions increased from 291·5 million in 2000 to 474·4 in 2018, representing a 62·7% increase. 14 countries accounted for nearly 70% of overcrowding in 2018 and nearly half of people living in overcrowded conditions resided in six countries. We also describe how the proportions and patterns of household overcrowding differ between and within countries and how these data have changed over the 19-year study period. The increases were likely to have been driven by booming urbanisation and population growth in Africa. Our work should be leveraged as a major opportunity to improve public health in Africa alongside broader human development goals. Understanding where a lack of adequate living space exists is also essential for monitoring the transmission of infectious diseases, such as SARS-CoV-2, as well as the spread of AMR, and could provide priority areas for benchmarking and introducing interventions for achieving SDG 11 in Africa.

## Data sharing

A detailed table of data sources is provided in the appendix (pp 5–27). All code used for these analyses is publicly available online at http://ghdx.healthdata.org/.

## Declaration of interests

All authors declare no competing interests.
