# Supplementary material for: Mapping local variation in household overcrowding across Africa from 2000 to 2018: a modelling study
Source: Lancet Planet Health. 2022 Aug 3;6(8):e670–81. doi: 10.1016/S2542-5196(22)00149-8 (PMC9364142; doi:10.1016/S2542-5196(22)00149-8)
Supplement: Supplementary appendix [file mmc1.pdf]

THE LANCET
Planetary Health

Supplementary appendix

This appendix formed part of the original submission and has been peer reviewed. We post it as supplied by the authors.

Supplement to: Chipeta MG, Kumaran EPA, Browne AJ, et al. Mapping local variation in household overcrowding across Africa from 2000 to 2018: a modelling study. *Lancet Planet Health* 2022; **6:** e670–81.

Mapping local variation in household overcrowding across Africa from 2000 to 2018: a modelling study – supplementary information.

Table of Contents

Figures ... ***1***

Tables ... ***1***

Household overcrowding definition ... ***2***

Household overcrowding data ... ***2***

2.1 Data extraction ... **2**

2.2 Polygon resampling ... **27**

2.3 Data preparation ... **27**

Household overcrowding model ... ***27***

3.1 Covariate selection ... **28**

3.2 Stacked ensemble model ... **29**

3.3 Geostatistical model ... **29**

3.3.1 Model specification ... 29

3.3.2 Spatial Mesh creation ... 30

3.4 Model fitting and validation ... **31**

Household overcrowding model results ... ***32***

4.1 Model results ... **32**

4.2 Model validation ... **35**

Gather Checklist ... ***38***

References ... ***39***

Figures

Figure S1: Map of data availability for household overcrowding model. ... 3

Figure S2: Plot of data availability for household overcrowding by country and year. ... 4

Figure S3: Polygon resampling. ... 27

Figure S4: Geographical regions for the model of household overcrowding. ... 28

Figure S5**:** Model covariates selection for each modelling region. ... 29

Figure S6**:** Finite element mesh for the Central sub-Saharan Africa region. ... 31

Figure S7: Five yearly estimates of the proportions of household overcrowding in Africa, at the administrative level 2 (district). ... 32

Figure S8: Five yearly estimates of change in the proportions of household overcrowding in Africa, at 5 x 5 km pixel level. ... 33

Figure S9: Estimates of the household overcrowding proportions in Africa for 2018 with the mean and upper and lower 95% uncertainty intervals. ... 34

Figure S10: Overlapping population–weighted quartiles of household overcrowding and relative uncertainty in 2018. ... 35

Figure S11: Validation plots for the model of household overcrowding proportions. ... 36

Figure S12: Validation plots for the model of household overcrowding for each modelling region. ... 37

Tables

Table S1: Surveys included in the household overcrowding model. ... 4

Table S2: Covariates included in the household overcrowding model. ... 28

Table S3: In- and out-of-sample metrics for the five-fold cross validation models on household overcrowding. ... 35

Table S4: GATHER Checklist. A checklist identifying each point of the Guidelines for Accurate and Transparent Health Estimates Reporting and whether they are covered in this study. 38

Household overcrowding definition

Household overcrowding is, in general, defined as a condition where the number of individuals occupying a dwelling exceeds the capacity of the dwelling space available. A number of definitions and metrices have been used to define overcrowding.[1,2] The most notable are: 1) the UN Habitat where overcrowding occurs when there are more than two people per habitable room (excluding bathrooms but including kitchens and living rooms)[3] 2) the United States Department of Housing and Urban Development (HUD) considers a household overcrowded if more than one person shares a room[1] and 3) the UK government defines a household as overcrowded if it has fewer bedrooms than it needs to avoid undesirable sharing, based on the age, sex and relationship of household members.[4] In the current study, we defined and created a binary outcome indicator of overcrowding as a ratio of individuals to sleeping rooms/spaces higher than two,[5] referred to as person-per-room (PPR).

Household overcrowding data

2.1 Data extraction

We assembled 386 surveys (USAID's Demographic Health Surveys (DHS), UNICEF's Multiple Indicator Cluster Surveys (MICS), IPUMS' population censuses and other country specific surveys) with information on household size and/or number of habitable rooms in a household based across Africa. Of these, 287 surveys had usable information from which data were extracted, covering 78,695,991 households between 2000 and 2018, from 51 African countries (Figures S1 and S2). Details of the surveys included in the analysis can be found in table S1. We extracted data on the number of habitable rooms in each household and the number of people reported to have slept in the house the night before the survey. Only surveys linkable to sub-national locations (either longitudes and latitudes or administrative divisions) were extracted. The geostatistical model utilised point locations directly and administrative divisions were linked to polygons in shapefiles, which were resampled to point locations, see Section 2.2 below. Shapefiles were obtained from the Food and Agriculture Organisations Global Administrative Unit Layers (GAUL; http://www.fao.org/geonetwork/), the Database of Global Administrative Areas (GADM; https://gadm.org/), and additional freely available sources online. When required, shapefiles were edited using R and ArcGIS to represent the district boundaries at the time the survey was conducted.

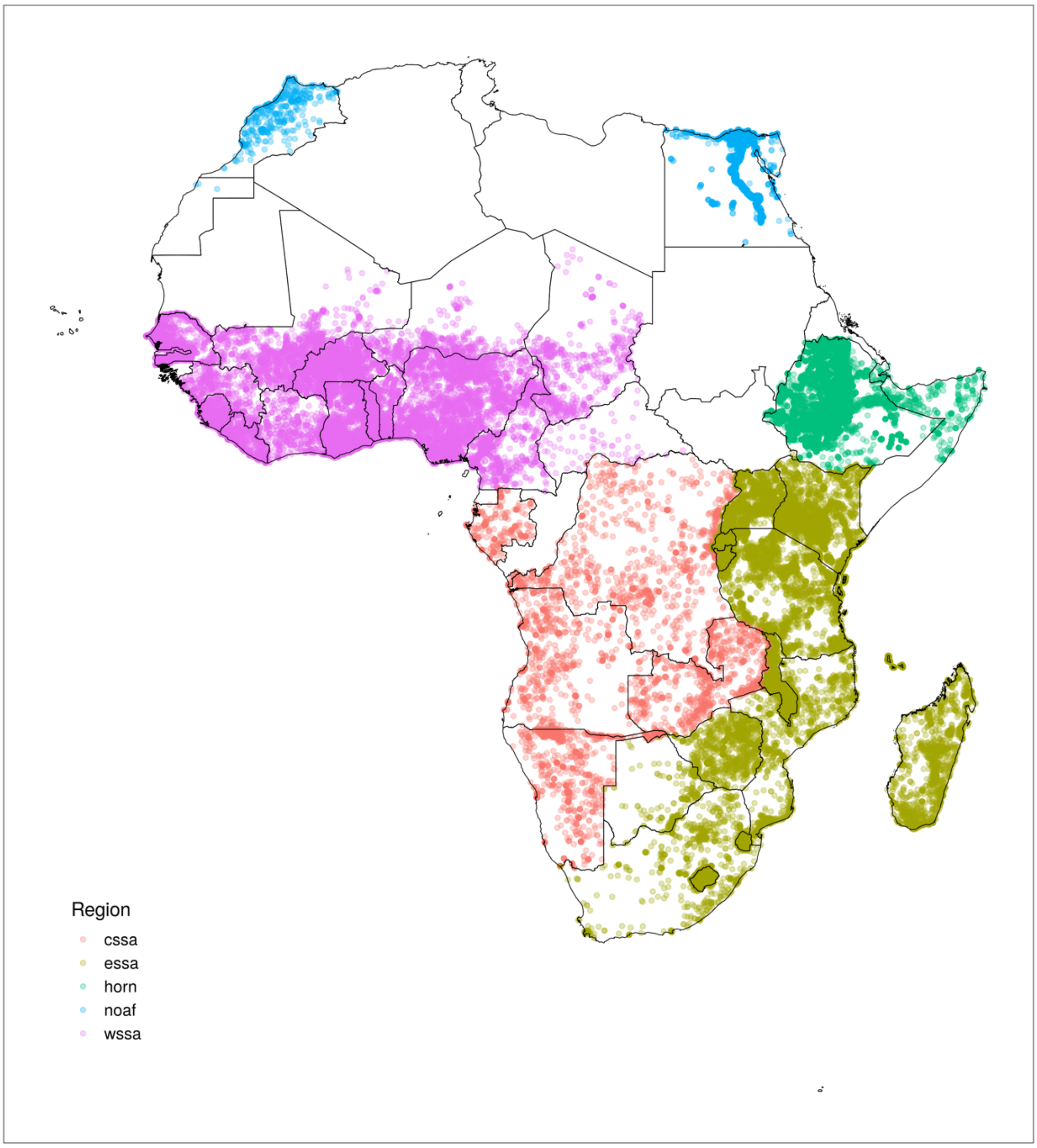

Figure S1: Map of data availability for household overcrowding model. **Countries with household surveys contributing to the model are coloured by region.**

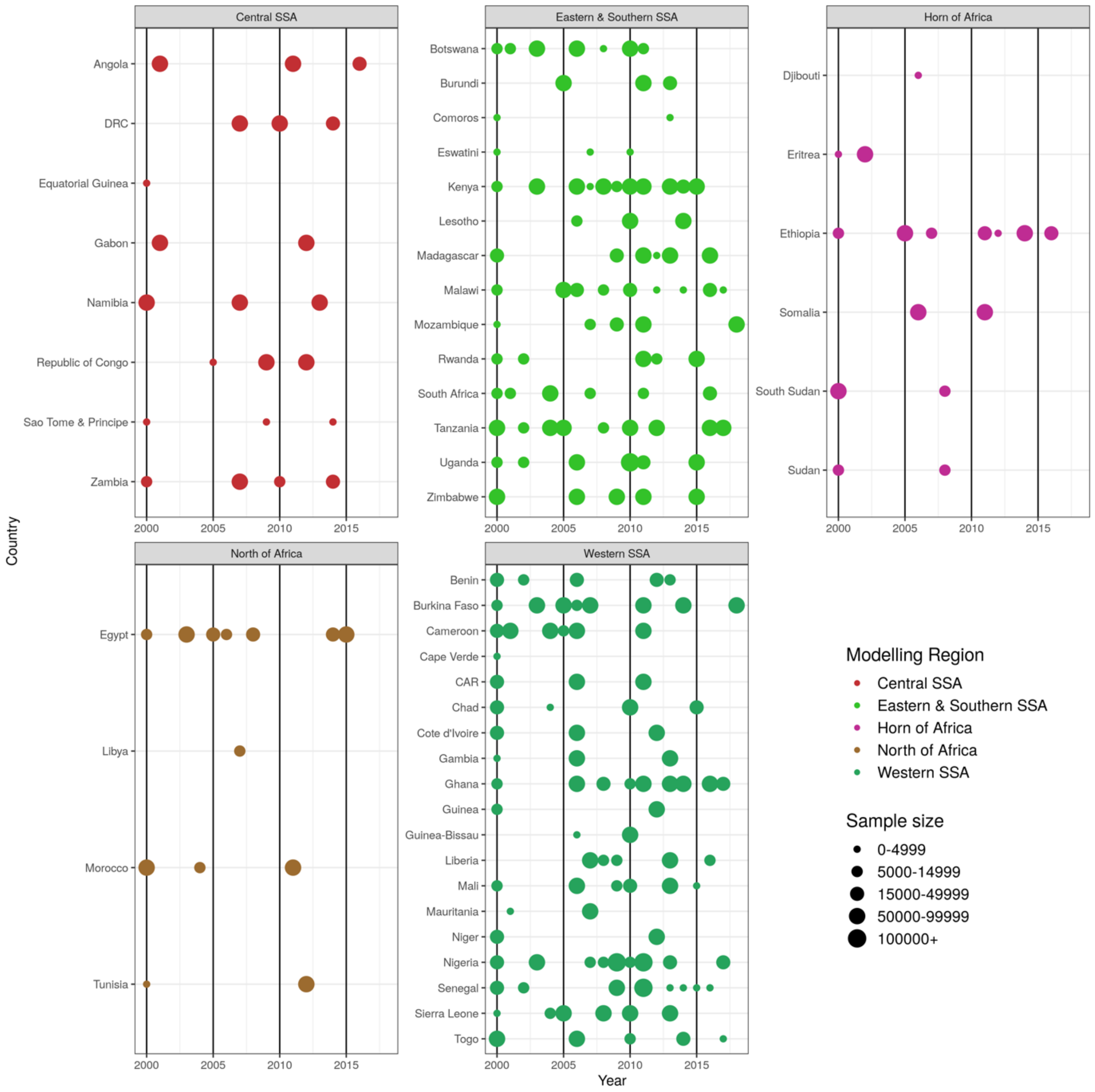

Figure S2: Plot of data availability for household overcrowding by country and year. The x-axis indicates the year and the y-axis the country in which the survey was undertaken**.** The size of the points is representative of the number of households in each survey with larger points containing more data, and the colour indicates the modelling region to which that country belongs.

Table S1: Surveys included in the household overcrowding model.

**Country**	**Year**	**Source**	**Citation**
Angola	2016	Demographic and Health Survey (DHS)	ICF International, Ministry of Health (Angola), National Institute of Statistics (Angola), United Nations Children's Fund (UNICEF). Angola Demographic and Health Survey 2015-2016. Fairfax, United States of America: ICF International, 2017.
Angola	2011	Demographic and Health Survey (DHS)	COSEP-Consulting Ltd., Consaúde Ltd., ICF International, National Malaria Control (Angola), President's Malaria Initiative (PMI). Angola Malaria Indicator Survey 2011. Fairfax, United States of America: ICF International.

Angola	2001	Multiple Indicator Cluster Survey (MICS)	National Institute of Statistics (Angola), United Nations Children's Fund (UNICEF). Angola Multiple Indicator Cluster Survey 2001. New York, United States of America: United Nations Children's Fund (UNICEF).
Benin	2012	Demographic and Health Survey (DHS)	ICF International, National Institute of Statistics and Economic Analysis (INSAE) (Benin), National Program Against AIDS (PNLS) (Benin). Benin Demographic and Health Survey 2011-2012. Fairfax, United States of America: ICF International, 2014.
Benin	2006	Demographic and Health Survey (DHS)	Macro International, Inc, National Institute of Statistics and Economic Analysis (INSAE) (Benin), National Program Against AIDS (PNLS) (Benin). Benin Demographic and Health Survey 2006. Fairfax, United States of America: ICF International.
Benin	2001	Demographic and Health Survey (DHS)	National Institute of Statistics and Economic Analysis (INSAE) (Benin), ORC Macro. Benin Demographic and Health Survey 2001. Fairfax, United States of America: ICF International.
Benin	2002	Integrated Public Use Microdata Series (IPUMS Census)	National Institute of Statistics and Economic Analysis (INSAE) (Benin), Minnesota Population Centre. Benin Population and Housing Census 2002 from the Integrated Public Use Microdata Series, International. Minneapolis, MN: IPUMS, 2018. https://doi.org/10.18128/D020.V7.1
Benin	2013	Integrated Public Use Microdata Series (IPUMS Census)	National Institute of Statistics and Economic Analysis (INSAE) (Benin), Minnesota Population Centre. Benin Population and Housing Census 2013 from the Integrated Public Use Microdata Series, International. Minneapolis, MN: IPUMS, 2018. https://doi.org/10.18128/D020.V7.1
Botswana	2003	BWA/HOUSEHOLD_INCOME_AND_EXPENDITURE_SURVEY	Central Statistics Office (Botswana). Botswana Household Income and Expenditure Survey 2002-2003. Gaborone, Botswana: Central Statistics Office (Botswana).
Botswana	2006	BWA/DEMOGRAPHIC_SURVEY	Central Statistics Office (Botswana). Botswana Demographic Survey 2006. Gaborone, Botswana: Central Statistics Office (Botswana).
Botswana	2008	BWA/FAMILY_HEALTH_SURVEY	Central Statistics Office (Botswana). Botswana Family Health Survey 2007-2008. Gaborone, Botswana: Central Statistics Office (Botswana), 2009.
Botswana	2010	BWA_CWIS	Central Statistics Office (Botswana). Botswana Core Welfare Indicators Survey 2009-2010.
Botswana	2000	Integrated Public Use Microdata Series (IPUMS Census)	Central Statistics Office (Botswana), Minnesota Population Centre. Botswana Population and Housing Census 1991 from the Integrated Public Use Microdata Series, International. Minneapolis: University of Minnesota, 2017.
Botswana	2001	Integrated Public Use Microdata Series (IPUMS Census)	Central Statistics Office (Botswana), Minnesota Population Centre. Botswana Population and Housing Census 2001 from the Integrated Public Use Microdata Series, International. Minneapolis: University of Minnesota, 2017.
Botswana	2011	Integrated Public Use Microdata Series (IPUMS Census)	Central Statistics Office (Botswana), Minnesota Population Centre. Botswana Population and Housing Census 2011 from the Integrated Public Use Microdata Series, International. Minneapolis: University of Minnesota, 2017.

Burkina Faso	2018	Demographic and Health Survey (DHS)	ICF International, National Centre for Research and Training on Malaria (CNRFP) (Burkina Faso), National Institute of Statistics and Demography (Burkina Faso), National Program for the Fight Against Malaria (PNLP) (Burkina Faso). Burkina Faso Malaria Indicator Survey 2017-2018. Fairfax, United States of America: ICF International, 2018.
Burkina Faso	2014	Demographic and Health Survey (DHS)	ICF International, National Centre for Research and Training on Malaria (CNRFP) (Burkina Faso), National Institute of Statistics and Demography (Burkina Faso), National Program for the Fight Against Malaria (PNLP) (Burkina Faso). Burkina Faso Malaria Indicator Survey 2014. Fairfax, United States of America: ICF International, 2015.
Burkina Faso	2011	Demographic and Health Survey (DHS)	ICF Macro, Ministry of Health (Burkina Faso), National Institute of Statistics and Demography (Burkina Faso). Burkina Faso Demographic and Health Survey 2010-2011. Fairfax, United States of America: ICF International.
Burkina Faso	2000	Demographic and Health Survey (DHS)	Macro International, Inc, National Institute of Statistics and Demography (Burkina Faso). Burkina Faso Demographic and Health Survey 1998-1999. Fairfax, United States of America: ICF International.
Burkina Faso	2000	Integrated Public Use Microdata Series (IPUMS Census)	Minnesota Population Centre, National Institute of Statistics and Demography (Burkina Faso). Burkina Faso Population and Housing Census 1996 from the Integrated Public Use Microdata Series, International: [Machine-readable database]. Minneapolis: University of Minnesota, 2013
Burkina Faso	2006	Integrated Public Use Microdata Series (IPUMS Census)	Minnesota Population Centre, National Institute of Statistics and Demography (Burkina Faso). Burkina Faso Population and Housing Census 2006 from the Integrated Public Use Microdata Series, International: [Machine-readable database]. Minneapolis: University of Minnesota, 2013.
Burkina Faso	2006	Multiple Indicator Cluster Survey (MICS)	National Institute of Statistics and Demography (Burkina Faso), United Nations Children's Fund (UNICEF). Burkina Faso Multiple Indicator Cluster Survey 2006. New York, United States of America: United Nations Children's Fund (UNICEF).
Burkina Faso	2003	World Bank Core Welfare Indicators Questionnaire (WB CWIQ)	National Institute of Statistics and Demography (Burkina Faso), World Bank. Burkina Faso Core Welfare Indicators Questionnaire Survey 2003. Ouagadougou, Burkina Faso: National Institute of Statistics and Demography (Burkina Faso).
Burkina Faso	2005	World Bank Core Welfare Indicators Questionnaire (WB CWIQ)	National Institute of Statistics and Demography (Burkina Faso), World Bank. Burkina Faso Core Welfare Indicators Questionnaire Survey 2005. Ouagadougou, Burkina Faso: National Institute of Statistics and Demography (Burkina Faso).
Burkina Faso	2007	World Bank Core Welfare Indicators Questionnaire (WB CWIQ)	National Institute of Statistics and Demography (INSD). Burkina Faso Core Welfare Indicators Questionnaire Survey 2007. Ouagadougou, Burkina Faso: National Institute of Statistics and Demography (INSD), 2008.
Burundi	2013	Demographic and Health Survey (DHS)	Burundi Institute of Statistics and Economic Studies, ICF Macro, Ministry of Public Health and the Fight Against AIDS (Burundi), National Institute of Public Health (Burundi). Burundi Malaria Indicator Survey 2012-2013. Fairfax, United States of America: ICF International, 2013.
Burundi	2011	Demographic and Health Survey (DHS)	Burundi Institute of Statistics and Economic Studies, ICF International, Ministry of Public Health and the Fight Against AIDS (Burundi). Burundi Demographic and Health Survey 2010-2011. Fairfax, United States of America: ICF International, 2012.
Burundi	2005	Multiple Indicator Cluster Survey (MICS)	United Nations Children's Fund (UNICEF), Burundi Institute of Statistics and Economic Studies, United Nations Population Fund (UNFPA). Burundi Multiple Indicator Cluster Survey 2005. New York, United States: United Nations Children's Fund (UNICEF).

Cameroon	2000	CMR/HH_SURVEY	National Institute of Statistics (Cameroon). Cameroon Household Survey 1996. Yaoundé, Cameroon: National Institute of Statistics (Cameroon).
Cameroon	2011	Demographic and Health Survey (DHS)	ICF International, Ministry of Economy, Planning and Regional Development (Cameroon), Ministry of Public Health (Cameroon), National Institute of Statistics (Cameroon), Pasteur Centre of Cameroon. Cameroon Demographic and Health Survey 2011. Fairfax, United States of America: ICF International.
Cameroon	2004	Demographic and Health Survey (DHS)	Macro International, Inc, National Institute of Statistics (Cameroon). Cameroon Demographic and Health Survey 2004. Fairfax, United States of America: ICF International.
Cameroon	2000	Demographic and Health Survey (DHS)	Macro International, Inc, Ministry of Economy, Planning and Regional Development (Cameroon). Cameroon Demographic and Health Survey 1991. Fairfax, United States of America: ICF International.
Cameroon	2000	Demographic and Health Survey (DHS)	Central Bureau of the Census and Population Studies (Cameroon), Macro International, Inc. Cameroon Demographic and Health Survey 1998. Fairfax, United States of America: ICF International.
Cameroon	2001	CMR/HH_SURVEY	National Institute of Statistics (Cameroon), Directorate of Statistics and National Accounts, Ministry of Economics and Finance (Cameroon), AFRISTAT. Cameroon Household Survey 2001. Yaoundé, Cameroon: National Institute of Statistics (Cameroon).
Cameroon	2005	Integrated Public Use Microdata Series (IPUMS Census)	Minnesota Population Centre, National Institute of Statistics (Cameroon), Central Bureau of the Census and Population Studies (Cameroon). Cameroon Population and Housing Census 2005 from the Integrated Public Use Microdata Series, International: [Machine-readable database]. Minneapolis: University of Minnesota, 2013
Cameroon	2006	Multiple Indicator Cluster Survey (MICS)	United Nations Children's Fund (UNICEF), National Institute of Statistics (Cameroon). Cameroon Multiple Indicator Cluster Survey 2006. New York, United States: United Nations Children's Fund (UNICEF).
Cameroon	2000	Multiple Indicator Cluster Survey (MICS)	Directorate of Statistics and National Accounts, Ministry of Economics and Finance (Cameroon), United Nations Children's Fund (UNICEF). Cameroon Multiple Indicator Cluster Survey 2000. New York, United States of America: United Nations Children's Fund (UNICEF).
Cape Verde	2000	CDC_RHS	Cape Verde National Statistics Institute (INE) Division of Reproductive Health-Centres for Disease Control and Prevention (CDC). (2000) Cape Verde Reproductive Health Survey 1998. Atlanta, United States: Centres for Disease Control and Prevention (CDC).
CAR	2000	Demographic and Health Survey (DHS)	Division of Statistics and Economic and Social Studies (Central African Republic), Macro International, Inc. Central African Republic Demographic and Health Survey 1994-1995. Fairfax, United States of America: ICF International.
CAR	2011	Multiple Indicator Cluster Survey (MICS)	Central African Institute of Statistics, Economic and Social Studies (ICASEES) (Central African Republic), ICF International. Central African Republic Multiple Indicator Cluster Survey 2010-2011. Fairfax, United States of America: ICF International, 2013.
CAR	2006	Multiple Indicator Cluster Survey (MICS)	United Nations Children's Fund (UNICEF). Central African Republic Multiple Indicator Cluster Survey 2006. New York, United States: United Nations Children's Fund (UNICEF).

CAR	2000	Multiple Indicator Cluster Survey (MICS)	Division of Statistics and Economic and Social Studies (Central African Republic), Ministry of Economy, Planning and International Cooperation (Central African Republic), United Nations Children's Fund (UNICEF). Central African Republic Multiple Indicator Cluster Survey 2000. New York, United States of America: United Nations Children's Fund (UNICEF).
Chad	2015	Demographic and Health Survey (DHS)	ICF International, National Institute for Statistics, Economic and Demographic Studies (INSEED) (Chad). Chad Demographic and Health Survey 2014-2015. Fairfax, United States of America: ICF International, 2016.
Chad	2004	Demographic and Health Survey (DHS)	Macro International, Inc, National Institute for Statistics, Economic and Demographic Studies (INSEED) (Chad). Chad Demographic and Health Survey 2004. Fairfax, United States of America: ICF International.
Chad	2000	Demographic and Health Survey (DHS)	Census Bureau (Chad), Macro International, Inc, National Institute for Statistics, Economic and Demographic Studies (INSEED) (Chad). Chad Demographic and Health Survey 1996-1997. Fairfax, United States of America: ICF International.
Chad	2010	Multiple Indicator Cluster Survey (MICS)	Ministry of Planning, Economy, and International Cooperation (Chad), National Institute for Statistics, Economic and Demographic Studies (INSEED) (Chad), United Nations Children's Fund (UNICEF). Chad Multiple Indicator Cluster Survey 2010. New York, United States of America: United Nations Children's Fund (UNICEF), 2014.
Chad	2000	Multiple Indicator Cluster Survey (MICS)	United Nations Children's Fund (UNICEF), Census Bureau (Chad), National Institute of Statistical, Economic and Demographic Studies (Chad). Chad Multiple Indicator Cluster Survey 2000. New York, United States: United Nations Children's Fund (UNICEF).
Comoros	2013	Demographic and Health Survey (DHS)	General Directorate of Statistics and Forecasting (Comoros), ICF International. Comoros Demographic and Health Survey 2012-2013. Fairfax, United States of America: ICF International.
Comoros	2000	Demographic and Health Survey (DHS)	Macro International, Inc, National Centre of Documentation and Scientific Research (Comoros). Comoros Demographic and Health Survey 1996. Fairfax, United States of America: ICF International.
Cote d'Ivoire	2012	Demographic and Health Survey (DHS)	ICF International, Ministry of the Fight Against AIDS (Côte d'Ivoire), National Institute of Statistics (Côte d'Ivoire). Côte d'Ivoire Demographic and Health Survey 2011-2012. Fairfax, United States of America: ICF International.
Cote d'Ivoire	2000	Demographic and Health Survey (DHS)	Macro International, Inc, National Institute of Statistics (Côte d'Ivoire). Côte d'Ivoire Demographic and Health Survey 1994. Fairfax, United States of America: ICF International.
Cote d'Ivoire	2000	Demographic and Health Survey (DHS)	Macro International, Inc, National Institute of Statistics (Côte d'Ivoire). Côte d'Ivoire Demographic and Health Survey 1998-1999. Fairfax, United States of America: ICF International.
Cote d'Ivoire	2006	Multiple Indicator Cluster Survey (MICS)	United Nations Children's Fund (UNICEF), National Institute of Statistics (Côte d'Ivoire). Côte d'Ivoire Multiple Indicator Cluster Survey 2006. New York, United States: United Nations Children's Fund (UNICEF).
Cote d'Ivoire	2000	Multiple Indicator Cluster Survey (MICS)	National School for Statistics and Economics Applied (ENSEA), United Nations Children's Fund (UNICEF), United Nations Educational, Scientific and Cultural Organization (UNESCO). Côte d'Ivoire Multiple Indicator Cluster Survey 2000. New York, United States of America: United Nations Children's Fund (UNICEF).

Djibouti	2006	Multiple Indicator Cluster Survey (MICS)	Ministry of Economy, Finance, and Planning in charge of Privatization (Djibouti), Ministry of Health (Djibouti), United Nations Children's Fund (UNICEF). Djibouti Multiple Indicator Cluster Survey 2006. New York, United States of America: United Nations Children's Fund (UNICEF).
DRC	2014	Demographic and Health Survey (DHS)	ICF International, Ministry of Planning and Monitoring Implementation of the Revolution of Modernity (Congo, DR), Ministry of Public Health (Congo, DR), National Institute of Statistics (Congo, DR). Democratic Republic of the Congo Demographic and Health Survey 2013-2014. Fairfax, United States of America: ICF International, 2014.
DRC	2007	Demographic and Health Survey (DHS)	Macro International, Inc, Ministry of Planning (Congo, DR). Democratic Republic of the Congo Demographic and Health Survey 2007. Fairfax, United States of America: ICF International.
DRC	2010	Multiple Indicator Cluster Survey (MICS)	National Statistical Institute (Congo, DR), Ministry of Planning (Congo, DR), United Nations Children's Fund (UNICEF). Congo, DR Multiple Indicator Cluster Survey 2010. New York, United States: United Nations Children's Fund (UNICEF).
Egypt	2015	Demographic and Health Survey (DHS)	El-Zanaty and Associates, ICF International, Ministry of Health and Population (Egypt), Population Council (Egypt). Egypt Special Demographic and Health Survey 2015. Fairfax, United States of America: ICF International.
Egypt	2014	Demographic and Health Survey (DHS)	El-Zanaty and Associates, ICF International, Ministry of Health and Population (Egypt). Egypt Demographic and Health Survey 2014. Fairfax, United States of America: ICF International.
Egypt	2008	Demographic and Health Survey (DHS)	El-Zanaty and Associates, Macro International, Inc, Ministry of Health and Population (Egypt). Egypt Demographic and Health Survey 2008. Fairfax, United States of America: ICF International.
Egypt	2005	Demographic and Health Survey (DHS)	El-Zanaty and Associates, Macro International, Inc, Ministry of Health and Population (Egypt), Population Council (Egypt). Egypt Demographic and Health Survey 2005. Fairfax, United States of America: ICF International.
Egypt	2003	Demographic and Health Survey (DHS)	El-Zanaty and Associates, Macro International, Inc, Ministry of Health and Population (Egypt), Population Council (Egypt). Egypt Interim Demographic and Health Survey 2003. Fairfax, United States of America: ICF International.
Egypt	2000	Demographic and Health Survey (DHS)	Macro International, Inc, Population Council (Egypt). Egypt Demographic and Health Survey 1992-1993. Fairfax, United States of America: ICF International.
Egypt	2000	Demographic and Health Survey (DHS)	Macro International, Inc, Population Council (Egypt). Egypt Demographic and Health Survey 1995-1996. Fairfax, United States of America: ICF International.
Egypt	2000	Demographic and Health Survey (DHS)	Macro International, Inc, Population Council (Egypt). Egypt Demographic and Health Survey 2000. Fairfax, United States of America: ICF International.
Egypt	2000	Integrated Public Use Microdata Series (IPUMS Census)	Central Agency for Public Mobilization and Statistics (CAPMAS) (Egypt), Minnesota Population Centre, University of Minnesota. Egypt Population, Housing, and Establishment Census 1996 - IPUMS. Minneapolis, United States of America: University of Minnesota.

Egypt	2006	Integrated Public Use Microdata Series (IPUMS Census)	Central Agency for Public Mobilization and Statistics (CAPMAS) (Egypt), Minnesota Population Centre, University of Minnesota. Egypt General Census for Population, Housing, and Establishments 2006 - IPUMS. Minneapolis, United States of America: University of Minnesota, 2011.
Equatorial Guinea	2000	Multiple Indicator Cluster Survey (MICS)	Ministry of Planning, Economic Development and Public Investment (Equatorial Guinea), United Nations Children's Fund (UNICEF). Equatorial Guinea Multiple Indicator Cluster Survey 2000. New York, United States of America: United Nations Children's Fund (UNICEF).
Eritrea	2002	Demographic and Health Survey (DHS)	Macro International, Inc, National Statistics and Evaluation Office (Eritrea). Eritrea Demographic and Health Survey 2002. Fairfax, United States of America: ICF International.
Eritrea	2000	Demographic and Health Survey (DHS)	Macro International, Inc, National Statistics Office (Eritrea). Eritrea Demographic and Health Survey 1995-1996. Fairfax, United States of America: ICF International.
Eswatini	2007	Demographic and Health Survey (DHS)	Central Statistical Office (Swaziland), Macro International, Inc. Swaziland Demographic and Health Survey 2006-2007. Fairfax, United States of America: ICF International.
Eswatini	2010	Multiple Indicator Cluster Survey (MICS)	Central Statistical Office (Swaziland), United Nations Children's Fund (UNICEF). Swaziland Multiple Indicator Cluster Survey 2010. New York, United States of America: United Nations Children's Fund (UNICEF).
Eswatini	2000	Multiple Indicator Cluster Survey (MICS)	Central Statistical Office (Swaziland), United Nations Children's Fund (UNICEF). Swaziland Multiple Indicator Cluster Survey 2000. New York, United States of America: United Nations Children's Fund (UNICEF).
Ethiopia	2016	Demographic and Health Survey (DHS)	Central Statistical Agency (Ethiopia), ICF International. Ethiopia Demographic and Health Survey 2016. Fairfax, United States of America: ICF International, 2017.
Ethiopia	2011	Demographic and Health Survey (DHS)	Central Statistical Agency (Ethiopia), ICF Macro, Ministry of Health (Ethiopia). Ethiopia Demographic and Health Survey 2010-2011. Fairfax, United States of America: ICF International.
Ethiopia	2005	Demographic and Health Survey (DHS)	Macro International, Inc, Population and Housing Census Commissions Office (PHCCO). Ethiopia Demographic and Health Survey 2005. Fairfax, United States of America: ICF International.
Ethiopia	2000	Demographic and Health Survey (DHS)	Central Statistical Agency (Ethiopia), ORC Macro. Ethiopia Demographic and Health Survey 2000. Calverton, United States of America: ORC Macro, 2001.
Ethiopia	2000	Integrated Public Use Microdata Series (IPUMS Census)	Minnesota Population Centre, Ethiopia Central Statistical Agency. Ethiopia Population and Housing Census 1994 from the Integrated Public Use Microdata Series, International: [Machine-readable database]. Minneapolis: University of Minnesota, 2015.
Ethiopia	2007	Integrated Public Use Microdata Series (IPUMS Census)	Minnesota Population Centre, Ethiopia Central Statistical Agency. Ethiopia Population and Housing Census 2007 from the Integrated Public Use Microdata Series, International: [Machine-readable database]. Minneapolis: University of Minnesota, 2015.

Ethiopia	2012	World Bank Living Standards Measurements Study (WB LSMS)	Central Statistical Agency (Ethiopia), World Bank. Ethiopia Rural Socioeconomic Survey 2011-2012. Washington DC, United States of America: World Bank.
Ethiopia	2014	World Bank Living Standards Measurements Study (WB LSMS)	Central Statistical Agency (Ethiopia), World Bank. Ethiopia Socioeconomic Survey 2013-2014. Washington DC, United States of America: World Bank, 2015.
Ethiopia	2016	World Bank Living Standards Measurements Study (WB LSMS)	Central Statistical Agency (Ethiopia), World Bank. Ethiopia Socioeconomic Survey 2015-2016. Washington DC, United States of America: World Bank, 2015.
Ethiopia	2016	ETH/WELFARE_MONITORING_SURVEY	Central Statistical Agency (Ethiopia). Ethiopia Welfare Monitoring Survey 2015-2016.
Gabon	2012	Demographic and Health Survey (DHS)	General Directorate of Statistics (Gabon), ICF International, Ministry of Economy, Employment and Sustainable Development (Gabon), Ministry of Health (Gabon). Gabon Demographic and Health Survey 2012. Fairfax, United States of America: ICF International, 2013.
Gabon	2001	Demographic and Health Survey (DHS)	General Directorate of Statistics and Economic Studies (Gabon), Macro International, Inc. Gabon Demographic and Health Survey 2000-2001. Fairfax, United States of America: ICF International.
Gambia	2013	Demographic and Health Survey (DHS)	Gambia Bureau of Statistics (GBOS), ICF International, Ministry of Health and Social Welfare (The Gambia). Gambia Demographic and Health Survey 2013. Fairfax, United States of America: ICF International, 2015.
Gambia	2006	Multiple Indicator Cluster Survey (MICS)	Gambia Bureau of Statistics (GBOS), United Nations Children's Fund (UNICEF). Gambia Multiple Indicator Cluster Survey 2005-2006. New York, United States of America: United Nations Children's Fund (UNICEF).
Gambia	2000	Multiple Indicator Cluster Survey (MICS)	Central Statistics Department (Gambia), United Nations Children's Fund (UNICEF). Gambia Multiple Indicator Cluster Survey 2000. New York, United States: United Nations Children's Fund (UNICEF).
Ghana	2017	Demographic and Health Survey (DHS)	Ghana Health Service, Ghana Statistical Service, ICF International. Ghana Special Demographic and Health Survey 2017. Fairfax, United States of America: ICF International, 2018.
Ghana	2016	Demographic and Health Survey (DHS)	Ghana Health Service, Ghana Statistical Service, ICF International, National Malaria Control Programme (Ghana), National Public Health and Reference Laboratory (NHPRL) (Ghana). Ghana Malaria Indicator Survey 2016. Fairfax, United States of America: ICF International.
Ghana	2014	Demographic and Health Survey (DHS)	Ghana Health Service, Ghana Statistical Service, ICF International. Ghana Demographic and Health Survey 2014. Fairfax, United States of America: ICF International, 2016.
Ghana	2008	Demographic and Health Survey (DHS)	Ghana Health Service, Ghana Statistical Service, Macro International, Inc. Ghana Special Demographic and Health Survey 2007-2008. Fairfax, United States of America: ICF International.

Ghana	2008	Demographic and Health Survey (DHS)	Ghana Statistical Service, Macro International, Inc, Ministry of Health (Ghana). Ghana Demographic and Health Survey 2008. Fairfax, United States of America: ICF International.
Ghana	2000	Demographic and Health Survey (DHS)	Ghana Statistical Service, Macro International, Inc. Ghana Demographic and Health Survey 1993-1994. Fairfax, United States of America: ICF International.
Ghana	2000	Demographic and Health Survey (DHS)	Ghana Statistical Service, Macro International, Inc. Ghana Demographic and Health Survey 1998-1999. Fairfax, United States of America: ICF International.
Ghana	2000	Integrated Public Use Microdata Series (IPUMS Census)	Ghana Statistical Service, Minnesota Population Centre. Ghana Population and Housing Census 2000 from the Integrated Public Use Microdata Series, International: [Machine-readable database]. Minneapolis: University of Minnesota.
Ghana	2010	Integrated Public Use Microdata Series (IPUMS Census)	Ghana Statistical Service, Minnesota Population Centre. Ghana Census 2010 from the Integrated Public Use Microdata Series, International: [Machine-readable database]. Minneapolis: University of Minnesota.
Ghana	2011	Multiple Indicator Cluster Survey (MICS)	Centres for Disease Control and Prevention (CDC), Ghana Statistical Service, Government of Japan, ICF Macro, Ministry of Health (Ghana), Navrongo Health Research Centre, United Nations Children's Fund (UNICEF), United Nations Population Fund (UNFPA), United States Agency for International Development (USAID). Ghana Multiple Indicator Cluster Survey 2011. New York, United States of America: United Nations Children's Fund (UNICEF), 2013.
Ghana	2011	Multiple Indicator Cluster Survey (MICS)	Institute of Statistical, Social and Economic Research, University of Ghana, United Nations Children's Fund (UNICEF). Ghana - Accra Multiple Indicator Cluster Survey 2010-2011. New York, United States of America: United Nations Children's Fund (UNICEF), 2014.
Ghana	2006	Multiple Indicator Cluster Survey (MICS)	Ministry of Health (MOH) (Ghana), Ghana Statistical Service and United Nations Children's Fund (UNICEF). Ghana Multiple Indicator Cluster Survey 2006. New York, United States: United Nations Children's Fund (UNICEF).
Ghana	2000	World Bank Living Standards Measurements Study (WB LSMS)	Ghana Statistical Service. Ghana Living Standards Survey 1998-1999.
Ghana	2013	World Bank Living Standards Measurements Study (WB LSMS)	Ghana Statistical Service, World Bank. Ghana Living Standards Measurement Survey 2012-2013. Accra, Ghana: Ghana Statistical Service.
Guinea	2012	Demographic and Health Survey (DHS)	ICF Macro, Ministry of Health and Public Hygiene (Guinea), National Institute of Statistics (Guinea). Guinea Demographic and Health Survey 2012. Fairfax, United States of America: ICF International.
Guinea	2000	Demographic and Health Survey (DHS)	Macro International, Inc, National Statistics Directorate (Guinea). Guinea Demographic and Health Survey 1999. Fairfax, United States of America: ICF International.
Guinea	2000	Integrated Public Use Microdata Series (IPUMS Census)	National Statistics Directorate (Guinea), Ministry of Economy, Finance, and Planning (Guinea), Minnesota Population Centre. Guinea General Census of Population and Housing 1996 from the Integrated Public Use Microdata Series, International: [Machine-readable database]. Minneapolis: University of Minnesota.

Guinea-Bissau	2010	Multiple Indicator Cluster Survey (MICS)	Centres for Disease Control and Prevention (CDC), National Statistics Institute (Guinea-Bissau), United Nations Children's Fund (UNICEF). Guinea-Bissau Multiple Indicator Cluster Survey 2010. New York, United States of America: United Nations Children's Fund (UNICEF), 2018.
Guinea-Bissau	2006	Multiple Indicator Cluster Survey (MICS)	United Nations Children's Fund (UNICEF), Government of Guinea-Bissau. Guinea-Bissau Multiple Indicator Cluster Survey 2006. New York, United States: United Nations Children's Fund (UNICEF).
Kenya	2000	KEN/WELFARE_MONITORING_SURVEY	Central Bureau of Statistics (Kenya). Kenya Welfare Monitoring Survey III 1997. Nairobi, Kenya: Kenya National Bureau of Statistics.
Kenya	2006	KEN/KIHBS	Central Bureau of Statistics (Kenya), UK Department for International Development (DFID), United States Agency for International Development (USAID), European Union (EU), Danish International Development Agency (DANIDA), World Bank (WB), United Nations Development Programme (UNDP). Kenya Integrated Household Budget Survey 2005-2006. Nairobi, Kenya: Central Bureau of Statistics (Kenya).
Kenya	2015	Demographic and Health Survey (DHS)	ICF International, Kenya National Bureau of Statistics, National Malaria Control Program (NMCP) (Kenya). Kenya Malaria Indicator Survey 2015. Fairfax, United States of America: ICF International, 2015.
Kenya	2014	Demographic and Health Survey (DHS)	ICF International, Kenya Medical Research Institute (KEMRI), Kenya National Bureau of Statistics, Ministry of Health (Kenya), National AIDS Control Council (Kenya), National Council for Population and Development (Kenya). Kenya Demographic and Health Survey 2014. Fairfax, United States of America: ICF International.
Kenya	2010	Demographic and Health Survey (DHS)	Centres for Disease Control and Prevention (CDC), ICF Macro, Kenya Medical Research Institute (KEMRI), Kenya National Bureau of Statistics, Measure DHS, Ministry of Public Health and Sanitation (Kenya), Population Services International (PSI), President's Malaria Initiative (PMI), United Nations Children's Fund (UNICEF), Walter Reed Project, World Health Organization (WHO). Kenya Malaria Indicator Survey 2010. Nairobi, Kenya: Kenya National Bureau of Statistics.
Kenya	2009	Demographic and Health Survey (DHS)	ICF Macro, Kenya Medical Research Institute (KEMRI), Kenya National Bureau of Statistics, Ministry of Public Health and Sanitation (Kenya), National AIDS and STI Control Programme (NASCOP) (Kenya), National Aids Control Council (NACC), National Coordinating Agency for Population and Development (Kenya). Kenya Demographic and Health Survey 2008-2009. Fairfax, United States of America: ICF International.
Kenya	2003	Demographic and Health Survey (DHS)	Centres for Disease Control and Prevention (CDC), Central Bureau of Statistics (Kenya), Macro International, Inc, Ministry of Health (Kenya), National Council for Population and Development (Kenya). Kenya Demographic and Health Survey 2003. Fairfax, United States of America: ICF International.
Kenya	2000	Demographic and Health Survey (DHS)	Central Bureau of Statistics (Kenya), Macro International, Inc, National Council for Population Development (NCPD). Kenya Demographic and Health Survey 1993. Fairfax, United States of America: ICF International.
Kenya	2000	Demographic and Health Survey (DHS)	Central Bureau of Statistics (Kenya), Macro International, Inc, National Council for Population Development (NCPD). Kenya Demographic and Health Survey 1998. Fairfax, United States of America: ICF International.

Kenya	2000	Integrated Public Use Microdata Series (IPUMS Census)	Central Bureau of Statistics (CBS) (Kenya), Minnesota Population Centre. Kenya Population and Housing Census 1999 from the Integrated Public Use Microdata Series, International: [Machine-readable database]. Minneapolis: University of Minnesota.
Kenya	2009	Integrated Public Use Microdata Series (IPUMS Census)	Minnesota Population Centre, Kenya National Bureau of Statistics. Kenya Population Census 2009 from the Integrated Public Use Microdata Series, International: [Machine-readable database]. Minneapolis: University of Minnesota, 2013.
Kenya	2014	Multiple Indicator Cluster Survey (MICS)	Kenya National Bureau of Statistics, Population Studies and Research Institute, University of Nairobi (Kenya), United Nations Children's Fund (UNICEF). Kenya - Bungoma County Multiple Indicator Survey 2013-2014. New York, United States of America: United Nations Children's Fund (UNICEF), 2015.
Kenya	2014	Multiple Indicator Cluster Survey (MICS)	Kenya National Bureau of Statistics, Population Studies and Research Institute, University of Nairobi (Kenya), United Nations Children's Fund (UNICEF). Kenya - Kakamega County Multiple Indicator Survey 2013-2014. New York, United States of America: United Nations Children's Fund (UNICEF), 2015.
Kenya	2014	Multiple Indicator Cluster Survey (MICS)	Kenya National Bureau of Statistics, Population Studies and Research Institute, University of Nairobi (Kenya), United Nations Children's Fund (UNICEF). Kenya - Turkana County Multiple Indicator Survey 2013-2014. New York, United States of America: United Nations Children's Fund (UNICEF), 2015.
Kenya	2011	Multiple Indicator Cluster Survey (MICS)	Kenya National Bureau of Statistics, United Nations Children's Fund (UNICEF). Kenya - Nyanza Province Multiple Indicator Cluster Survey 2011. Nairobi, Kenya: Kenya National Bureau of Statistics.
Kenya	2008	Multiple Indicator Cluster Survey (MICS)	Kenya National Bureau of Statistics, United Nations Children's Fund (UNICEF). Kenya - Eastern Province Multiple Indicator Cluster Survey 2008. Nairobi, Kenya: Kenya National Bureau of Statistics.
Kenya	2007	Multiple Indicator Cluster Survey (MICS)	Kenya National Bureau of Statistics, United Nations Children's Fund (UNICEF). Kenya - North Eastern Province Multiple Indicator Cluster Survey 2007. Nairobi, Kenya: Kenya National Bureau of Statistics.
Kenya	2000	Multiple Indicator Cluster Survey (MICS)	Central Bureau of Statistics (Kenya), United Nations Children's Fund (UNICEF). Kenya Multiple Indicator Cluster Survey 2000. New York, United States: United Nations Children's Fund (UNICEF).
Kenya	2013	KEN/AIDS_INDICATOR_SURVEY	Kenya National Bureau of Statistics, Ministry of Devolution and Planning (Kenya), Ministry of Health (Kenya), National AIDS and STI Control Programme (NASCOP) (Kenya). Kenya AIDS Indicator Survey 2012-2013. Nairobi, Kenya: Kenya National Bureau of Statistics.
Lesotho	2014	Demographic and Health Survey (DHS)	ICF International, Ministry of Health and Social Welfare (Lesotho). Lesotho Demographic and Health Survey 2014. Fairfax, United States of America: ICF International.
Lesotho	2010	Demographic and Health Survey (DHS)	ICF Macro, Ministry of Health and Social Welfare (Lesotho). Lesotho Demographic and Health Survey 2009-2010. Fairfax, United States of America: ICF International.
Lesotho	2006	Integrated Public Use Microdata Series (IPUMS Census)	Bureau of Statistics (Lesotho), Minnesota Population centre. Lesotho Population and Housing Census 2006 from the Integrated Public Use Microdata Series, International. Minneapolis, MN: IPUMS, 2018. https://doi.org/10.18128/D020.V7.1

Liberia	2016	Demographic and Health Survey (DHS)	ICF International, Liberia Institute for Statistics and Geo-information Services (LISGIS), National Malaria Control Program (Liberia). Liberia Malaria Indicator Survey 2016. Fairfax, United States of America: ICF International, 2017.
Liberia	2013	Demographic and Health Survey (DHS)	ICF International, Liberia Institute for Statistics and Geo-information Services (LISGIS), National AIDS and STI Control Program (NACP), Ministry of Health and Social Welfare (Liberia). Liberia Demographic and Health Survey 2013. Fairfax, United States of America: ICF International.
Liberia	2009	Demographic and Health Survey (DHS)	ICF Macro, Liberia Institute for Statistics and Geo-information Services (LISGIS), National Malaria Control Program (Liberia). Liberia Malaria Indicator Survey 2008-2009. Fairfax, United States of America: ICF International.
Liberia	2007	Demographic and Health Survey (DHS)	Liberia Institute for Statistics and Geo-information Services (LISGIS), Macro International, Inc. Liberia Demographic and Health Survey 2006-2007. Fairfax, United States of America: ICF International.
Liberia	2008	Integrated Public Use Microdata Series (IPUMS Census)	Liberia Institute for Statistics and Geo-information Services (LISGIS), Minnesota Population Centre. Liberia Census 2008 from the Integrated Public Use Microdata Series, International: [Machine-readable database]. Minneapolis: University of Minnesota.
Libya	2007	Pan Arab Project for Family Health (PAPFAM)	League of Arab States, National Centre for Disease Control (Libya), Pan Arab Project for Family Health (PAPFAM). Libya Family Health Survey 2007.
Madagascar	2016	Demographic and Health Survey (DHS)	ICF International, Ministry of Public Health (Madagascar), National Institute of Statistics (Madagascar), National Program for the Fight Against Malaria (PNLP) (Madagascar), Pasteur Institute of Madagascar (IPM). Madagascar Malaria Indicator Survey 2016. Fairfax, United States of America: ICF International, 2017.
Madagascar	2013	Demographic and Health Survey (DHS)	ICF International, National Institute of Statistics (Madagascar), National Program for the Fight Against Malaria (PNLP) (Madagascar), Pasteur Institute of Madagascar (IPM). Madagascar Malaria Indicator Survey 2013. Fairfax, United States of America: ICF International, 2013.
Madagascar	2011	Demographic and Health Survey (DHS)	ICF International, National Institute of Statistics (Madagascar), National Program for the Fight Against Malaria (PNLP) (Madagascar), Pasteur Institute of Madagascar (IPM). Madagascar Malaria Indicator Survey 2011. Fairfax, United States of America: ICF International.
Madagascar	2009	Demographic and Health Survey (DHS)	ICF Macro, National Institute of Statistics (Madagascar). Madagascar Demographic and Health Survey 2008-2009. Fairfax, United States of America: ICF International.
Madagascar	2000	Demographic and Health Survey (DHS)	Department of Applied Research for Development (Madagascar), Macro International, Inc. Madagascar Demographic and Health Survey 1992. Calverton, United States of America: Macro International, Inc.
Madagascar	2000	Demographic and Health Survey (DHS)	Macro International, Inc, National Institute of Statistics (Madagascar). Madagascar Demographic and Health Survey 1997. Fairfax, United States of America: ICF International.
Madagascar	2012	Multiple Indicator Cluster Survey (MICS)	National Institute of Statistics (Madagascar), United Nations Children's Fund (UNICEF). Madagascar - South Multiple Indicator Cluster Survey 2012. New York, United States of America: United Nations Children's Fund (UNICEF), 2015.

Madagascar	2000	Multiple Indicator Cluster Survey (MICS)	National Institute of Statistics (Madagascar), United Nations Children's Fund (UNICEF). Madagascar Multiple Indicator Cluster Survey 2000. New York, United States of America: United Nations Children's Fund (UNICEF).
Malawi	2017	Demographic and Health Survey (DHS)	ICF International, National Malaria Control Program (Malawi). Malawi Malaria Indicator Survey 2017. Fairfax, United States of America: ICF International, 2018.
Malawi	2016	Demographic and Health Survey (DHS)	Emory University and Centres for Disease Control & Prevention Collaboration, ICF International, Ministry of Health (Malawi), National Statistical Office of Malawi. Malawi Demographic and Health Survey 2015-2016. Fairfax, United States of America: ICF International, 2017.
Malawi	2014	Demographic and Health Survey (DHS)	ICF International, Ministry of Health (Malawi), National Malaria Control Program (Malawi), National Statistical Office of Malawi. Malawi Malaria Indicator Survey 2014. Fairfax, United States of America: ICF International, 2015.
Malawi	2012	Demographic and Health Survey (DHS)	ICF International, National Malaria Control Program (Malawi). Malawi Malaria Indicator Survey 2012. Fairfax, United States of America: ICF International.
Malawi	2010	Demographic and Health Survey (DHS)	ICF Macro, National Statistical Office of Malawi. Malawi Demographic and Health Survey 2010. Fairfax, United States of America: ICF International.
Malawi	2005	Demographic and Health Survey (DHS)	Macro International, Inc, National Statistical Office of Malawi. Malawi Demographic and Health Survey 2004-2005. Fairfax, United States of America: ICF International.
Malawi	2000	Demographic and Health Survey (DHS)	Macro International, Inc, National Statistical Office of Malawi. Malawi Demographic and Health Survey 2000. Fairfax, United States of America: ICF International.
Malawi	2000	Integrated Public Use Microdata Series (IPUMS Census)	National Statistical Office (Malawi), Minnesota Population Centre. Malawi Population and Housing Census 1998 from the Integrated Public Use Microdata Series, International: [Machine-readable database]. Minneapolis: University of Minnesota, 2011.
Malawi	2008	Integrated Public Use Microdata Series (IPUMS Census)	National Statistical Office (Malawi), Minnesota Population Centre. Malawi Population and Housing Census 2008 from the Integrated Public Use Microdata Series, International: [Machine-readable database]. Minneapolis: University of Minnesota, 2011.
Malawi	2006	Multiple Indicator Cluster Survey (MICS)	United Nations Children's Fund (UNICEF), National Statistics Office (Malawi). Malawi Multiple Indicator Cluster Survey 2006. New York, United States: United Nations Children's Fund (UNICEF).
Mali	2015	Demographic and Health Survey (DHS)	ICF International, INFO-STAT (Mali), Ministry of Health and Public Hygiene (Mali), National Institute of Public Health Research (INRSP) (Mali), National Institute of Statistics (INSTAT) (Mali), National Program for the Fight Against Malaria (Mali). Mali Malaria Indicator Survey 2015. Fairfax, United States of America: ICF International, 2016.
Mali	2013	Demographic and Health Survey (DHS)	ICF International, INFO-STAT (Mali), Ministry of Health (Mali), National Institute of Statistics (INSTAT) (Mali), Planning and Statistics Unit, Ministry of Health (Mali). Mali Demographic and Health Survey 2012-2013. Fairfax, United States of America: ICF International, 2014.

Mali	2010	Demographic and Health Survey (DHS)	ICF Macro, INFO-STAT (Mali), National Program for the Fight Against Malaria (Mali). Mali Special Demographic and Health Survey 2010. Fairfax, United States of America: ICF International, 2011.
Mali	2006	Demographic and Health Survey (DHS)	Macro International, Inc, Ministry of Health (Mali), National Directorate of Statistics and Informatics (DNSI) (Mali). Mali Demographic and Health Survey 2006. Fairfax, United States of America: ICF International.
Mali	2000	Demographic and Health Survey (DHS)	Macro International, Inc, National Directorate of Statistics and Informatics (DNSI) (Mali), Planning and Statistics Unit, Ministry of Health (Mali). Mali Demographic and Health Survey 2001. Fairfax, United States of America: ICF International.
Mali	2000	Integrated Public Use Microdata Series (IPUMS Census)	Central Census Bureau (Mali), Minnesota Population Centre. Mali General Population and Housing Census 1998 from the Integrated Public Use Microdata Series, International: [Machine-readable database]. Minneapolis: University of Minnesota.
Mali	2009	Integrated Public Use Microdata Series (IPUMS Census)	Central Census Bureau (Mali), Minnesota Population Centre. Mali Census 2009 from the Integrated Public Use Microdata Series, International: [Machine-readable database]. Minneapolis: University of Minnesota.
Mauritania	2001	Demographic and Health Survey (DHS)	Macro International, Inc, National Office of Statistics (Mauritania). Mauritania Demographic and Health Survey 2000-2001. Fairfax, United States of America: ICF International.
Mauritania	2007	Multiple Indicator Cluster Survey (MICS)	National Office of Statistics (Mauritania), United Nations Children's Fund (UNICEF). Mauritania Multiple Indicator Cluster Survey 2007. New York, United States of America: United Nations Children's Fund (UNICEF).
Morocco	2004	Demographic and Health Survey (DHS)	League of Arab States, Macro International, Inc, Ministry of Health (Morocco). Morocco Demographic and Health Survey 2003-2004. Fairfax, United States of America: ICF International.
Morocco	2000	Demographic and Health Survey (DHS)	Macro International, Inc, Ministry of Public Health (Morocco). Morocco Demographic and Health Survey 1992. Fairfax, United States of America: ICF International.
Morocco	2004	Integrated Public Use Microdata Series (IPUMS Census)	Minnesota Population Centre, High Commission for Planning (Morocco). Morocco Population and Housing Census 2004 from the Integrated Public Use Microdata Series, International: [Machine-readable database]. Minneapolis: University of Minnesota, 2012.
Morocco	2011	Pan Arab Project for Family Health (PAPFAM)	Ministry of Health (Morocco), Pan Arab Project for Family Health (PAPFAM), United Nations Children's Fund (UNICEF), United Nations Population Fund (UNFPA), World Health Organization (WHO). Morocco National Survey on Population and Family Health 2010-2011.
Mozambique	2018	Demographic and Health Survey (DHS)	Ministry of Health (Mozambique), National Institute of Statistics (INE) (Mozambique), ICF Macro, Mozambique Malaria Indicator Survey 2018. Fairfax, United States of America: ICF International.
Mozambique	2011	Demographic and Health Survey (DHS)	ICF Macro, Manhica Health Research Centre (CISM), Ministry of Health (Mozambique), National Institute of Statistics (INE) (Mozambique). Mozambique Demographic and Health Survey 2011. Fairfax, United States of America: ICF International.

Mozambique	2009	Demographic and Health Survey (DHS)	ICF Macro, Ministry of Health (Mozambique), National Institute of Statistics (INE) (Mozambique). Mozambique AIDS Indicator Survey 2009. Fairfax, United States of America: ICF International, 2010.
Mozambique	2000	Demographic and Health Survey (DHS)	Macro International, Inc, National Institute of Statistics (INE) (Mozambique). Mozambique Demographic and Health Survey 1997. Fairfax, United States of America: ICF International.
Mozambique	2007	Integrated Public Use Microdata Series (IPUMS Census)	Minnesota Population Centre, Mozambique National Statistics Institute. Mozambique Census 2007 from the Integrated Public Use Microdata Series, International: [Machine-readable database]. Minneapolis: University of Minnesota, 2015.
Mozambique	2009	Multiple Indicator Cluster Survey (MICS)	United Nations Children's Fund (UNICEF), National Statistics Institute (Mozambique). Mozambique Multiple Indicator Cluster Survey 2008-2009. New York, United States: United Nations Children's Fund (UNICEF).
Namibia	2013	Demographic and Health Survey (DHS)	ICF International, Ministry of Health and Social Services (Namibia), Namibia Institute of Pathology, Namibia Statistics Agency. Namibia Demographic and Health Survey 2013. Fairfax, United States of America: ICF International.
Namibia	2007	Demographic and Health Survey (DHS)	Macro International, Inc, Ministry of Health and Social Services (Namibia). Namibia Demographic and Health Survey 2006-2007. Fairfax, United States of America: ICF International.
Namibia	2000	Demographic and Health Survey (DHS)	Central Statistics Office (Namibia), Macro International, Inc, Ministry of Health and Social Services (Namibia). Namibia Demographic and Health Survey 1992. Fairfax, United States of America: ICF International.
Namibia	2000	Demographic and Health Survey (DHS)	Macro International, Inc, Ministry of Health and Social Services (Namibia), National Planning Commission (Namibia). Namibia Demographic and Health Survey 2000. Fairfax, United States of America: ICF International.
Niger	2012	Demographic and Health Survey (DHS)	ICF International, Ministry of Public Health (Niger), National Institute of Statistics (Niger). Niger Demographic and Health Survey 2012. Fairfax, United States of America: ICF International.
Niger	2000	Demographic and Health Survey (DHS)	Department of Statistics and National Accounts (Niger), Macro International, Inc. Niger Demographic and Health Survey 1992. Fairfax, United States of America: ICF International.
Niger	2000	Demographic and Health Survey (DHS)	CARE International, Macro International, Inc. Niger Demographic and Health Survey 1998. Fairfax, United States of America: ICF International.
Niger	2000	Multiple Indicator Cluster Survey (MICS)	Government of Niger, Macro International, Inc, United Nations Children's Fund (UNICEF). Niger Multiple Indicator Cluster Survey 2000. New York, United States of America: United Nations Children's Fund (UNICEF).
Niger	2012	World Bank Living Standards Measurements Study (WB LSMS)	National Institute of Statistics (Niger), World Bank. Niger National Survey on Household Living Conditions and Agriculture 2011-2012.
Nigeria	2013	Demographic and Health Survey (DHS)	ICF International, National Population Commission of Nigeria. Nigeria Demographic and Health Survey 2013. Fairfax, United States of America: ICF International.

Nigeria	2010	Demographic and Health Survey (DHS)	ICF Macro, National Malaria Control Programme (Nigeria), National Population Commission of Nigeria. Nigeria Malaria Indicator Survey 2010. Fairfax, United States of America: ICF International.
Nigeria	2008	Demographic and Health Survey (DHS)	Macro International, Inc, National Population Commission of Nigeria. Nigeria Demographic and Health Survey 2008. Fairfax, United States of America: ICF International, 2009.
Nigeria	2003	Demographic and Health Survey (DHS)	Department for International Development (DFiD) (United Kingdom), National Population Commission of Nigeria, ORC Macro, United Nations Children's Fund (UNICEF), United Nations Population Fund (UNFPA). Nigeria Demographic and Health Survey 2003. Fairfax, United States of America: ICF International.
Nigeria	2000	Demographic and Health Survey (DHS)	Macro International, Inc, National Population Commission of Nigeria. Nigeria Demographic and Health Survey 1999. Calverton, United States of America: Macro International, Inc.
Nigeria	2007	Integrated Public Use Microdata Series (IPUMS Census)	National Bureau of Statistics (Nigeria), Minnesota Population Centre. Nigeria General Household Survey 2007 from the Integrated Public Use Microdata Series, International: [Machine-readable database]. Minneapolis: University of Minnesota.
Nigeria	2008	Integrated Public Use Microdata Series (IPUMS Census)	National Bureau of Statistics (Nigeria), Minnesota Population Centre. Nigeria General Household Survey 2008 from the Integrated Public Use Microdata Series, International: [Machine-readable database]. Minneapolis: University of Minnesota.
Nigeria	2009	Integrated Public Use Microdata Series (IPUMS Census)	National Bureau of Statistics (Nigeria), Minnesota Population Centre. Nigeria General Household Survey 2009 from the Integrated Public Use Microdata Series, International: [Machine-readable database]. Minneapolis: University of Minnesota.
Nigeria	2010	Integrated Public Use Microdata Series (IPUMS Census)	National Bureau of Statistics (Nigeria), Minnesota Population Centre. Nigeria General Household Survey 2010 from the Integrated Public Use Microdata Series, International: [Machine-readable database]. Minneapolis: University of Minnesota.
Nigeria	2011	Integrated Public Use Microdata Series (IPUMS Census)	National Bureau of Statistics (Nigeria), Minnesota Population Centre. Nigeria General Household Survey 2010-2011 from the Integrated Public Use Microdata Series, International: [Machine-readable database]. Minneapolis: University of Minnesota.
Nigeria	2017	Multiple Indicator Cluster Survey (MICS)	National Agency for the Control of AIDS (Nigeria), National Bureau of Statistics (Nigeria), National Primary Health Care Development Agency (NPHCDA) (Nigeria), United Nations Children's Fund (UNICEF). Nigeria Multiple Indicator Cluster Survey with National Immunization Coverage Survey Supplement 2016-2017. New York, United States of America: United Nations Children's Fund (UNICEF), 2018.
Nigeria	2011	Multiple Indicator Cluster Survey (MICS)	National Bureau of Statistics (Nigeria), United Nations Children's Fund (UNICEF). Nigeria Multiple Indicator Cluster Survey 2011. New York, United States of America: United Nations Children's Fund (UNICEF), 2013.
Nigeria	2007	Multiple Indicator Cluster Survey (MICS)	United Nations Children's Fund (UNICEF), National Bureau of Statistics (Nigeria). Nigeria Multiple Indicator Cluster Survey 2007. New York, United States: United Nations Children's Fund (UNICEF).

Nigeria	2000	Multiple Indicator Cluster Survey (MICS)	National Bureau of Statistics (Nigeria), United Nations Children's Fund (UNICEF). Nigeria Multiple Indicator Cluster Survey 1999. Abuja, Nigeria: National Bureau of Statistics (Nigeria).
Republic of Congo	2012	Demographic and Health Survey (DHS)	ICF International, Ministry of Health (Congo, Rep.), National Centre for Statistics and Economic Studies (Congo, Rep.). Congo Demographic and Health Survey 2011-2012. Fairfax, United States of America: ICF International.
Republic of Congo	2009	Demographic and Health Survey (DHS)	Macro International, Inc, National Centre for Statistics and Economic Studies (Congo, Rep.). Congo Demographic and Health Survey 2005. Fairfax, United States of America: ICF International.
Republic of Congo	2005	Demographic and Health Survey (DHS)	Macro International, Inc, National Centre for Statistics and Economic Studies (Congo, Rep.). Congo Demographic and Health Survey 2005. Fairfax, United States of America: ICF International.
Rwanda	2015	Demographic and Health Survey (DHS)	ICF International, Ministry of Health (Rwanda), National Institute of Statistics of Rwanda. Rwanda Demographic and Health Survey 2014-2015. Fairfax, United States of America: ICF International, 2016.
Rwanda	2011	Demographic and Health Survey (DHS)	ICF Macro, Ministry of Health (Rwanda), National Institute of Statistics of Rwanda. Rwanda Demographic and Health Survey 2010-2011. Fairfax, United States of America: ICF International.
Rwanda	2011	Demographic and Health Survey (DHS)	ICF International, Joint United Nations Program on HIV/AIDS (UNAIDS), National Institute of Statistics of Rwanda, Rwanda Biomedical Centre/Institute of HIV/AIDS, Disease Control and Prevention Department, School of Public Health, University of Rwanda. Rwanda Special Demographic and Health Survey 2011. Fairfax, United States of America: ICF International, 2012.
Rwanda	2000	Demographic and Health Survey (DHS)	Macro International, Inc, National Office of Population (Rwanda). Rwanda Demographic and Health Survey 2000. Fairfax, United States of America: ICF International.
Rwanda	2000	Integrated Public Use Microdata Series (IPUMS Census)	National Census Commission (Rwanda), Minnesota Population Centre. Rwanda Population and Housing Census 1991 from the Integrated Public Use Microdata Series, International: [Machine-readable database]. Minneapolis: University of Minnesota.
Rwanda	2002	Integrated Public Use Microdata Series (IPUMS Census)	National Census Commission (Rwanda), Minnesota Population Centre. Rwanda Population and Housing Census 2002 from the Integrated Public Use Microdata Series, International: [Machine-readable database]. Minneapolis: University of Minnesota.
Rwanda	2012	Integrated Public Use Microdata Series (IPUMS Census)	National Institute of Statistics (Rwanda), Minnesota Population Centre. Rwanda Population and Housing Census 2012 from the Integrated Public Use Microdata Series, International. Minneapolis, MN: IPUMS, 2018. https://doi.org/10.18128/D020.V7.1
Rwanda	2012	Rwanda Census	National Institute of Statistics of Rwanda. Rwanda Population and Housing Census 2012. Kigali, Rwanda: National Institute of Statistics of Rwanda, 2015.
Rwanda	2000	Multiple Indicator Cluster Survey (MICS)	Department of Statistics (Rwanda), United Nations Children's Fund (UNICEF). Rwanda Multiple Indicator Cluster Survey 2000. New York, United States: United Nations Children's Fund (UNICEF).

Sao Tome & Principe	2009	Demographic and Health Survey (DHS)	ICF Macro, Ministry of Health (Sao Tome and Principe), National Institute of Statistics (Sao Tome and Principe). Sao Tome and Principe Demographic and Health Survey 2008-2009. Fairfax, United States of America: ICF International.
Sao Tome & Principe	2014	Multiple Indicator Cluster Survey (MICS)	Global Fund to Fight Aids Tuberculosis and Malaria (GFATM), ICF International, National Centre for Endemic Diseases (CNE) (Sao Tome and Principe), National Institute of Statistics (Sao Tome and Principe), United Nations Children's Fund (UNICEF), United Nations Development Programme (UNDP). Sao Tome and Principe Multiple Indicator Cluster Survey 2014. New York, United States of America: United Nations Children's Fund (UNICEF), 2016.
Sao Tome & Principe	2000	Multiple Indicator Cluster Survey (MICS)	National Institute of Statistics (Sao Tome and Principe), United Nations Children's Fund (UNICEF). Sao Tome and Principe Multiple Indicator Cluster Survey 2000. New York, United States of America: United Nations Children's Fund (UNICEF).
Senegal	2016	Demographic and Health Survey (DHS)	ICF International, Ministry of Health and Social Action (Senegal), National Agency of Statistics and Demography (Senegal). Senegal Continuous Demographic and Health Survey 2016. Fairfax, United States of America: ICF International, 2017.
Senegal	2015	Demographic and Health Survey (DHS)	Cheikh Anta Diop University, ICF International, National Agency of Statistics and Demography (Senegal). Senegal Continuous Demographic and Health Survey 2015. Fairfax, United States of America: ICF International, 2016.
Senegal	2014	Demographic and Health Survey (DHS)	Cheikh Anta Diop University, ICF International, National Agency of Statistics and Demography (Senegal). Senegal Continuous Demographic and Health Survey 2014. Fairfax, United States of America: ICF International.
Senegal	2013	Demographic and Health Survey (DHS)	ICF International, Ministry of Health and Social Action (Senegal), National Agency of Statistics and Demography (Senegal). Senegal Continuous Demographic and Health Survey 2012-2013. Fairfax, United States of America: ICF International.
Senegal	2011	Demographic and Health Survey (DHS)	Centre for Research in Human Development (CRDH), Cheikh Anta Diop University, Hospital Aristide Le Dantec, ICF Macro, National Agency of Statistics and Demography (Senegal). Senegal Demographic and Health Survey 2010-2011. Fairfax, United States of America: ICF International.
Senegal	2009	Demographic and Health Survey (DHS)	Macro International, Inc, Research Centre for Human Development (Senegal). Senegal Malaria Indicator Survey 2008-2009. Fairfax, United States of America: ICF International.
Senegal	2000	Demographic and Health Survey (DHS)	Groupe SERDHA, Macro International, Inc, Ministry of Health and Prevention (Senegal). Senegal Demographic and Health Survey 1999-2000. Fairfax, United States of America: ICF International.
Senegal	2000	Demographic and Health Survey (DHS)	Directorate of Forecasting and Statistics, Ministry of the Economy, Finance and Planning (Senegal), Macro International, Inc. Senegal Demographic and Health Survey 1997. Fairfax, United States of America: ICF International.
Senegal	2000	Demographic and Health Survey (DHS)	Directorate of Forecasting and Statistics, Ministry of the Economy, Finance and Planning (Senegal), Macro International, Inc. Senegal Demographic and Health Survey 1992-1993. Fairfax, United States of America: ICF International.
Senegal	2000	Demographic and Health Survey (DHS)	Department of Statistics (Senegal), Westinghouse; Institute for Resource Development. Senegal Demographic and Health Survey 1986. Fairfax, United States of America: ICF International.

Senegal	2002	Integrated Public Use Microdata Series (IPUMS Census)	Directorate of Forecasting and Statistics (Senegal), Minnesota Population Centre. Senegal General Population and Housing Census 2002 from the Integrated Public Use Microdata Series, International: [Machine-readable database]. Minneapolis: University of Minnesota.
Sierra Leone	2013	Demographic and Health Survey (DHS)	ICF International, Ministry of Health and Sanitation (Sierra Leone), Statistics Sierra Leone. Sierra Leone Demographic and Health Survey 2013. Fairfax, United States of America: ICF International, 2014.
Sierra Leone	2008	Demographic and Health Survey (DHS)	Macro International, Inc, Statistics Sierra Leone. Sierra Leone Demographic and Health Survey 2008. Fairfax, United States of America: ICF International.
Sierra Leone	2004	Integrated Public Use Microdata Series (IPUMS Census)	Statistics Sierra Leone and Minnesota Population Centre. Sierra Leone Population and Housing Census 2004 from the Integrated Public Use Microdata Series, International: [Machine-readable database]. Minneapolis: University of Minnesota, 2011.
Sierra Leone	2010	Multiple Indicator Cluster Survey (MICS)	Statistics Sierra Leone, United Nations Children's Fund (UNICEF). Sierra Leone Multiple Indicator Cluster Survey 2010. New York, United States of America: United Nations Children's Fund (UNICEF).
Sierra Leone	2005	Multiple Indicator Cluster Survey (MICS)	United Nations Children's Fund (UNICEF), Statistics Sierra Leone. Sierra Leone Multiple Indicator Cluster Survey 2005. New York, United States: United Nations Children's Fund (UNICEF).
Sierra Leone	2000	Multiple Indicator Cluster Survey (MICS)	Central Statistics Office (Sierra Leone), United Nations Children's Fund (UNICEF). Sierra Leone Multiple Indicator Cluster Survey 2000. New York, United States of America: United Nations Children's Fund (UNICEF).
Somalia	2011	Multiple Indicator Cluster Survey (MICS)	Ministry of National Planning and Development (Somaliland), United Nations Children's Fund (UNICEF). Somalia - Somaliland Multiple Indicator Cluster Survey 2011. New York, United States of America: United Nations Children's Fund (UNICEF), 2015.
Somalia	2011	Multiple Indicator Cluster Survey (MICS)	Puntland Ministry of Planning and International Cooperation (Somalia), United Nations Children's Fund (UNICEF). Somalia - Northeast Zone Multiple Indicator Cluster Survey 2011. New York, United States of America: United Nations Children's Fund (UNICEF), 2015.
Somalia	2006	Multiple Indicator Cluster Survey (MICS)	Pan Arab Project for Family Health (PAPFAM), United Nations Children's Fund (UNICEF). Somalia Multiple Indicator Cluster Survey 2006. New York, United States of America: United Nations Children's Fund (UNICEF).
South Africa	2016	Demographic and Health Survey (DHS)	Department of Health (South Africa), ICF International, South African Medical Research Council, Statistics South Africa. South Africa Demographic and Health Survey 2016. Fairfax, United States of America: ICF International, 2019.
South Africa	2004	Demographic and Health Survey (DHS)	Department of Health (South Africa), Macro International, Inc, South African Medical Research Council. South Africa Demographic and Health Survey 2003-2004.
South Africa	2000	Demographic and Health Survey (DHS)	Department of Health (South Africa), Macro International, Inc, South African Medical Research Council. South Africa Demographic and Health Survey 1998. Fairfax, United States of America: ICF International.

South Africa	2000	Integrated Public Use Microdata Series (IPUMS Census)	Central Statistical Service (South Africa), Minnesota Population Centre. South Africa Census 1996 from the Integrated Public Use Microdata Series, International: [Machine-readable database]. Minneapolis: University of Minnesota.
South Africa	2001	Integrated Public Use Microdata Series (IPUMS Census)	Statistics South Africa, Minnesota Population Centre. South Africa Census 2001 from the Integrated Public Use Microdata Series, International: [Machine-readable database]. Minneapolis: University of Minnesota.
South Africa	2007	Integrated Public Use Microdata Series (IPUMS Census)	Statistics South Africa, Minnesota Population Centre. South Africa Community Survey 2007 from the Integrated Public Use Microdata Series, International: [Machine-readable database]. Minneapolis: University of Minnesota.
South Africa	2011	Integrated Public Use Microdata Series (IPUMS Census)	Minnesota Population Centre, Statistics South Africa. South Africa Population and Housing Census 2011 from the Integrated Public Use Microdata Series, International: [Machine-readable database]. Minneapolis: University of Minnesota, 2015.
South Africa	2000	World Bank Living Standards Measurements Study (WB LSMS)	Southern Africa Labour Development Research Unit (SALDRU), University of Cape Town, World Bank. South Africa Living Standards Measurement Study 1993. Washington DC, United States of America: World Bank.
South Africa	2016	Republic of South Africa Household Survey (RSAHHS)	Statistics South Africa. South Africa General Household Survey 2016. Cape Town, South Africa: DataFirst, 2015.
South Africa	2000	Republic of South Africa Household Survey (RSAHHS)	Statistics South Africa. South Africa October Household Survey 1998.
South Africa	2000	Republic of South Africa Household Survey (RSAHHS)	Central Statistical Service (South Africa). South Africa October Household Survey 1996.
South Africa	2000	Republic of South Africa Household Survey (RSAHHS)	Central Statistical Service (South Africa). South Africa October Household Survey 1997.
South Sudan	2008	Integrated Public Use Microdata Series (IPUMS Census)	Minnesota Population Centre, Southern Sudan Centre for Census, Statistics and Evaluation. Sudan - South Sudan Population and Housing Census 2008 from the Integrated Public Use Microdata Series, International: [Machine-readable database]. Minneapolis: University of Minnesota, 2013
South Sudan	2000	Multiple Indicator Cluster Survey (MICS)	United Nations Children's Fund (UNICEF). South Sudan Multiple Indicator Cluster Survey 1999. New York, United States: United Nations Children's Fund (UNICEF).
Sudan	2008	Integrated Public Use Microdata Series (IPUMS Census)	National Population Census Council (Sudan), Central Bureau of Statistics (Sudan), Southern Sudan Centre for Census, Statistics and Evaluation (SSCCSE), Minnesota Population Centre. Sudan Population and Housing Census 2008 from the Integrated Public Use Microdata Series, International: [Machine-readable database]. Minneapolis: University of Minnesota, 2011.
Sudan	2000	Multiple Indicator Cluster Survey (MICS)	Central Bureau of Statistics (Sudan), Federal Ministry of Health (Sudan), United Nations Children's Fund (UNICEF). Sudan Multiple Indicator Cluster Survey 2000. New York, United States of America: United Nations Children's Fund (UNICEF).

Tanzania	2017	Demographic and Health Survey (DHS)	ICF International, Ministry of Health (Zanzibar), Ministry of Health, Community Development, Gender, Elderly and Children (MoHCDEC) (Tanzania), National Bureau of Statistics (Tanzania), Office of the Chief Government Statistician (OCGS) (Zanzibar). Tanzania Malaria Indicator Survey 2017. Fairfax, United States of America: ICF International, 2018.
Tanzania	2016	Demographic and Health Survey (DHS)	ICF International, Ministry of Health (Zanzibar), Ministry of Health, Community Development, Gender, Elderly and Children (MoHCDEC) (Tanzania), National Bureau of Statistics (Tanzania), Office of the Chief Government Statistician (OCGS) (Zanzibar). Tanzania Demographic and Health Survey 2015-2016. Fairfax, United States of America: ICF International, 2016.
Tanzania	2012	Demographic and Health Survey (DHS)	ICF International, National Bureau of Statistics (Tanzania), Office of the Chief Government Statistician (OCGS) (Zanzibar), Tanzania Commission for AIDS (TACAIDS), Zanzibar AIDS Commission (ZAC). Tanzania AIDS Indicator Survey 2011-2012. Fairfax, United States of America: ICF International, 2013.
Tanzania	2010	Demographic and Health Survey (DHS)	ICF Macro, National Bureau of Statistics (Tanzania). Tanzania Demographic and Health Survey 2009-2010. Fairfax, United States of America: ICF International.
Tanzania	2008	Demographic and Health Survey (DHS)	Macro International, Inc, National Bureau of Statistics (Tanzania), Office of the Chief Government Statistician (OCGS) (Zanzibar), Tanzania Commission for AIDS (TACAIDS), Zanzibar AIDS Commission (ZAC). Tanzania HIV/AIDS and Malaria Indicator Survey 2007-2008. Fairfax, United States of America: ICF International.
Tanzania	2005	Demographic and Health Survey (DHS)	Macro International, Inc, National Bureau of Statistics (Tanzania). Tanzania Demographic and Health Survey 2004-2005. Fairfax, United States of America: ICF International.
Tanzania	2004	Demographic and Health Survey (DHS)	National Bureau of Statistics (Tanzania), ORC Macro, Tanzania Commission for AIDS (TACAIDS). Tanzania AIDS Indicator Survey 2003-2004. Fairfax, United States of America: ICF International.
Tanzania	2000	Demographic and Health Survey (DHS)	Bureau of Statistics (Tanzania), Macro International, Inc, Planning Commission (Tanzania). Tanzania Demographic and Health Survey 1996. Fairfax, United States of America: ICF International.
Tanzania	2000	Demographic and Health Survey (DHS)	Macro International, Inc, National Bureau of Statistics (Tanzania). Tanzania Demographic and Health Survey 1999. Fairfax, United States of America: ICF International.
Tanzania	2002	Integrated Public Use Microdata Series (IPUMS Census)	National Bureau of Statistics (Tanzania), Minnesota Population Centre. Tanzania Population and Housing Census 2002 from the Integrated Public Use Microdata Series, International: [Machine-readable database]. Minneapolis: University of Minnesota.
Togo	2017	Demographic and Health Survey (DHS)	ICF International, National Institute of Hygiene, Ministry of Health (Togo), National Institute of Statistics and Economic and Demographic Studies (INSEED) (Togo), National Program to Fight Malaria, Ministry of Health (Togo). Togo Malaria Indicator Survey 2017. Fairfax, United States of America: ICF International, 2018.
Togo	2014	Demographic and Health Survey (DHS)	Directorate General of Statistics and National Accounts (Togo), ICF International, Ministry of Health (Togo), Ministry of Planning, Development and Zoning (Togo). Togo Demographic and Health Survey 2013-2014. Fairfax, United States of America: ICF International, 2015.

Togo	2000	Demographic and Health Survey (DHS)	Department of Statistics (Togo), Macro International, Inc. Togo Demographic and Health Survey 1998. Fairfax, United States of America: ICF International.
Togo	2010	Integrated Public Use Microdata Series (IPUMS Census)	Government of Togo, People's Republic of China, United Nations Children's Fund (UNICEF), United Nations Development Programme (UNDP), United Nations Population Fund (UNFPA). Togo Population and Housing Census 2010. Lomé, Togo: Directorate General of Statistics and National Accounts (Togo).
Togo	2010	Multiple Indicator Cluster Survey (MICS)	Directorate General of Statistics and National Accounting (Togo), United Nations Children's Fund (UNICEF). Togo Multiple Indicator Cluster Survey 2010. New York, United States: United Nations Children's Fund (UNICEF).
Togo	2006	Multiple Indicator Cluster Survey (MICS)	Directorate General of Statistics and National Accounting (Togo), United Nations Children's Fund (UNICEF). Togo Multiple Indicator Cluster Survey 2006. New York, United States: United Nations Children's Fund (UNICEF).
Tunisia	2000	Demographic and Health Survey (DHS)	Macro Systems, Inc.; Institute for Resource Development, National Office for Family and Population, Ministry of Public Health (Tunisia). Tunisia Demographic and Health Survey 1988. Fairfax, United States of America: ICF International.
Tunisia	2012	Multiple Indicator Cluster Survey (MICS)	Ministry of Regional Development and Planning (Tunisia), National Institute of Statistics (Tunisia), United Nations Children's Fund (UNICEF). Tunisia Multiple Indicator Cluster Survey 2011-2012. New York, United States of America: United Nations Children's Fund (UNICEF), 2014.
Uganda	2015	Demographic and Health Survey (DHS)	ICF International, Uganda Bureau of Statistics. Uganda Demographic and Health Survey 2016. Fairfax, United States of America: ICF International, 2018.
Uganda	2011	Demographic and Health Survey (DHS)	ICF Macro, Uganda Bureau of Statistics. Uganda Demographic and Health Survey 2011. Fairfax, United States of America: ICF International.
Uganda	2011	Demographic and Health Survey (DHS)	ICF Macro, Uganda Bureau of Statistics. Uganda Demographic and Health Survey 2011. Fairfax, United States of America: ICF International.
Uganda	2010	Demographic and Health Survey (DHS)	ICF Macro, Ministry of Health (Uganda), Mulago Hospital, Uganda Bureau of Statistics, United Nations Children's Fund (UNICEF), World Health Organization (WHO). Uganda Malaria Indicator Survey 2009-2010. Fairfax, United States of America: ICF International.
Uganda	2006	Demographic and Health Survey (DHS)	Macro International, Inc, Uganda Bureau of Statistics. Uganda Demographic and Health Survey 2006. Fairfax, United States of America: ICF International.
Uganda	2000	Demographic and Health Survey (DHS)	Macro International, Inc, Statistics Department (Uganda). Uganda Demographic and Health Survey 1995. Fairfax, United States of America: ICF International.
Uganda	2000	Integrated Public Use Microdata Series (IPUMS Census)	Uganda Bureau of Statistics, Minnesota Population Centre. Uganda Population and Housing Census 1991 from the Integrated Public Use Microdata Series, International: [Machine-readable database]. Minneapolis: University of Minnesota.

Uganda	2002	Integrated Public Use Microdata Series (IPUMS Census)	Uganda Bureau of Statistics, Minnesota Population Centre. Uganda Population and Housing Census 2002 from the Integrated Public Use Microdata Series, International: [Machine-readable database]. Minneapolis: University of Minnesota.
Uganda	2006	Uganda Household Survey	Economic Policy Research Centre (Uganda), Uganda Bureau of Statistics. Uganda Household Survey 2005-2006.
Zambia	2014	Demographic and Health Survey (DHS)	Central Statistical Office (Zambia), ICF International, Ministry of Health (Zambia), Tropical Diseases Research Centre, University Teaching Hospital (Zambia), University of Zambia. Zambia Demographic and Health Survey 2013-2014. Fairfax, United States of America: ICF International.
Zambia	2007	Demographic and Health Survey (DHS)	Central Statistical Office (Zambia), Macro International, Inc. Zambia Demographic and Health Survey 2007. Fairfax, United States of America: ICF International.
Zambia	2000	Demographic and Health Survey (DHS)	Central Statistical Office (Zambia), Macro International, Inc, University of Zambia. Zambia Demographic and Health Survey 1992. Fairfax, United States of America: ICF International.
Zambia	2000	Demographic and Health Survey (DHS)	Central Statistical Office (Zambia), Macro International, Inc, Ministry of Health (Zambia). Zambia Demographic and Health Survey 1996-1997. Fairfax, United States of America: ICF International.
Zambia	2000	Integrated Public Use Microdata Series (IPUMS Census)	Central Statistical Office (Zambia), Minnesota Population Centre. Zambia Census 2000 from the Integrated Public Use Microdata Series, International: [Machine-readable database]. Minneapolis: University of Minnesota.
Zambia	2000	Integrated Public Use Microdata Series (IPUMS Census)	Central Statistical Office (Zambia), Minnesota Population Centre. Zambia Census 1990 from the Integrated Public Use Microdata Series, International: [Machine-readable database]. Minneapolis: University of Minnesota.
Zambia	2010	Integrated Public Use Microdata Series (IPUMS Census)	Central Statistical Office (Zambia), Minnesota Population Centre. Zambia Census 2010 from the Integrated Public Use Microdata Series, International: [Machine-readable database]. Minneapolis: University of Minnesota.
Zambia	2010	ZMB/LCMS	Central Statistical Office (Zambia). Zambia Living Conditions Monitoring Survey 2010.
Zimbabwe	2015	Demographic and Health Survey (DHS)	ICF International, National Microbiology Reference Laboratory, Harare Central Hospital (NMRL) (Zimbabwe), Zimbabwe National Statistics Agency. Zimbabwe Demographic and Health Survey 2015. Fairfax, United States of America: ICF International, 2016.
Zimbabwe	2011	Demographic and Health Survey (DHS)	ICF Macro, Zimbabwe National Statistics Agency. Zimbabwe Demographic and Health Survey 2010-2011. Calverton, United States of America: ICF Macro, 2012.
Zimbabwe	2006	Demographic and Health Survey (DHS)	Central Statistical Office (Zimbabwe), Macro International, Inc. Zimbabwe Demographic and Health Survey 2005-2006. Fairfax, United States of America: ICF International.

Zimbabwe	2000	Demographic and Health Survey (DHS)	Central Statistical Office (Zimbabwe), Macro International, Inc. Zimbabwe Demographic and Health Survey 1999. Fairfax, United States of America: ICF International.
Zimbabwe	2009	Multiple Indicator Cluster Survey (MICS)	Central Statistical Office (Zimbabwe). Zimbabwe Multiple Indicator Monitoring Survey 2009. New York, United States of America: United Nations Children's Fund (UNICEF).

2.2 Polygon resampling

The methods for polygon resampling are depicted in figure S3. For each polygon we cropped a raster of the WorldPop global population estimates[6], with a cell area of 5 x 5–km at the equator, to that area (figure S3A). We sampled 10,000–point locations (longitude and latitude of the cell centroid) from each polygon, with a sampling probability proportional to the cell population (figure S3B). *K*-means clustering was then applied to the point locations, so that there was one cluster per 1,000 raster cells within the polygon. Each cluster was then assigned an integration weight proportional of the number of point locations that were geographically closer to this cluster than any other (figure S3C). The sum of the weights of the point locations for each polygon resampled was equal to one. The resampled, weighted point locations were combined with the actual point data (which were assigned weights of one), and this dataset used as input data for the geostatistical model. In our dataset, over 95% of the data comprised of point level data, with polygon level data contributing less than 5% of the total data used in the analysis.

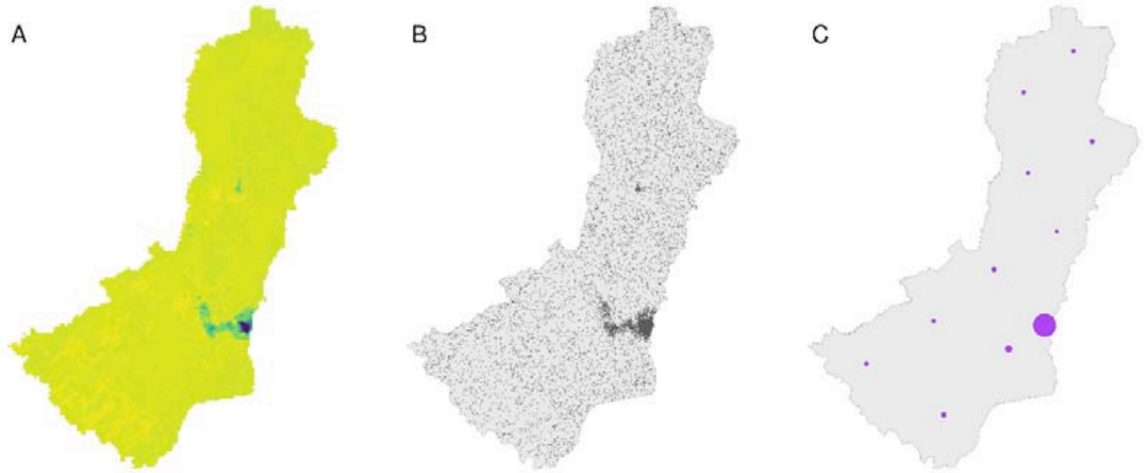

Figure S3: Polygon resampling. A pictorial representation of polygon resampling methods using k-means clustering. a) a raster of the total population cropped to the polygon to be resampled; b) 10,000–point locations sampled with a probability based on the cell population; c) k-means clustering applied to the point locations in (b), the size of the point represents the integration weight; (figure from Golding et al. 2017[7]).

2.3 Data preparation

The resampled, weighted point locations were linked to the data for that polygon and added to the data linked to actual point locations which were assigned a weight of one. This data was curated and used in the geospatial model. Data were collapsed to one data point per point location per year. Household overcrowding was defined following WHO guidance as having more than 2 people per habitable room[8].

Household overcrowding model

We fit a two-stage Bayesian binomial hierarchal model (see Section 3.3) to estimate the proportion of household overcrowding for each 5 x 5–km pixel for the 54 African countries. The model was fit separately for each region (figure S4) to improve computational stability and to incorporate overcrowding pattern differences between regions, and to allow modelling as well as assessment of differing effects and covariates' influence (see below,

covariates selection). Data for island countries was restricted to Madagascar, Cape Verde, Sao Tome & Principe islands and Comoros.

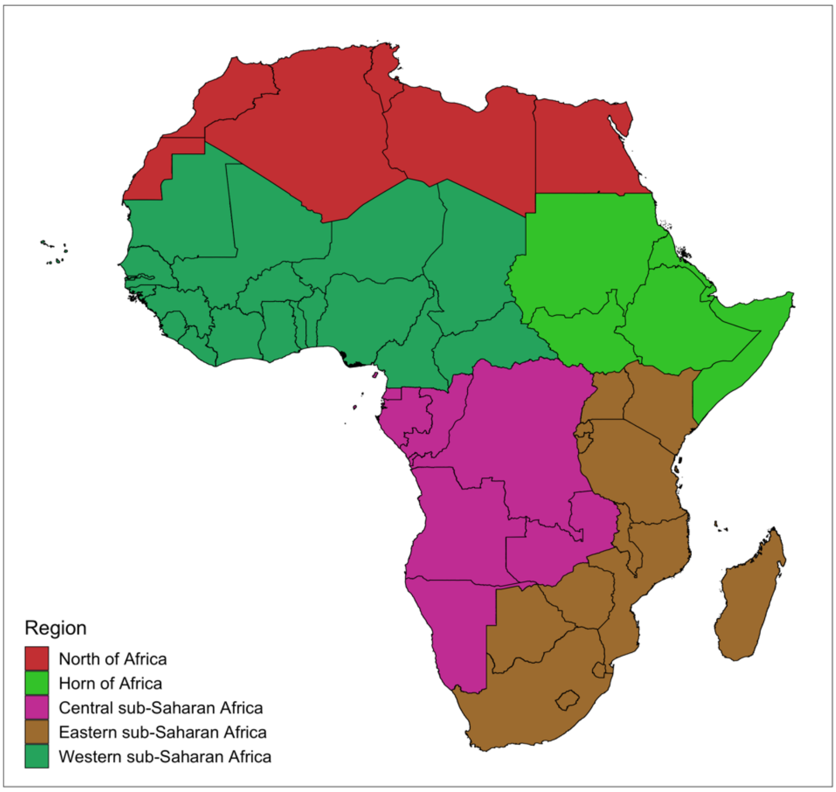

Figure S4: Geographical regions for the model of household overcrowding.

3.1 Covariate selection

The covariates that were chosen to inform the household overcrowding proportions were selected based on social-economic/environmental plausibility and importance in the model. Covariates were checked for correlation using the Pearson correlation coefficient, if one of any pair of covariates had an $r^2 > 0.8$ this was dropped from the analysis. covariates were then normalised by subtracting the mean from each value and dividing them by the standard deviation, resulting in all covariates having a mean of 0 and a standard deviation of 1. This is considered important in penalised regression models as it puts the regression coefficient onto the same scale and allows for the comparisons of covariates. Covariate selection was based on their importance in the child models and their impact on the predictive performance of the model. Final covariate selection was based on those informing the models with the best predictive validity. The final model was informed by those with the best out-of-sample metrics after running the model with five-fold cross-validation and covariates. The selected covariates are displayed in table S2 and figure S5 for each modelling region.

Table S2: Covariates included in the household overcrowding model.

**Covariate name**	**Covariate source**
Ratio of dependants to working-age adults	Christopher T. Lloyd, Alessandro Sorichetta & Andrew J. Tatem. High resolution global gridded data for use in population studies. Scientific Data 4, Article number: 170001 (2017) doi:10.1038/sdata.2017.1 http://www.worldpop.org.uk/data/get_data/
DPT3 vaccine coverage	Produced by the local burden of disease team at the Institute of Health Metrics and evaluation.
Education	Graetz, N. et al. Mapping local variation in educational attainment across Africa. Nature https://doi.org/10.1038/nature25761 (2018).

Gross domestic product (GDP) per pixel	Kummu, M., Taka, M., & Guillaume, J. H. A. Data Descriptor: Gridded global datasets for Gross Domestic Product and Human Development Index over 1990-2015. Nature: Scientific Data (2018).
Stunting	Produced by the local burden of disease team at the Institute of Health Metrics and evaluation.
Wasting	Produced by the local burden of disease team at the Institute of Health Metrics and evaluation.
Mean temperature	Harris, I., Jones, P. d., Osborn, T. j. & Lister, D. h. Updated high-resolution grids of monthly climatic observations – the CRU TS3.10 dataset. Int. J. Climatol. 34, 623–642 (2014).
Population	Lloyd, C. T., Sorichetta, A. & Tatem, A. J. High resolution global gridded data for use in population studies. Sci. Data 4, sdata20171 (2017). World Pop. Get data. Available at: http://www.worldpop.org.uk/data/get_data/.
Irrigation	The irrigation index of each pixel calculated by the local burden of disease team at the Institute of Health Metrics and evaluation.
Human Development Index (HDI)	The human development index of each pixel calculated by the local burden of disease team at the Institute of Health Metrics and evaluation.

3.2 Stacked ensemble model

In order to capture the effects of the covariates, we employed a stacked ensemble model[9]. This modelling strategy allows covariate selection, captures potential non-linear effects and accounts for interactions between covariates, while improving the predictive power over standalone models. For each region, we fit three different child models namely boosted regression trees (BRT), generalised additive models (GAM) and penalised regression model (elastic net), using five-fold cross-validation to produce out-of-sample predictions for each data point. The BRT, a decision tree-based machine learning algorithm, was fit using “xgboost” and the “caret” package version 6.085. A grid of potential model parameters were trialled and the final model fit on parameters resulting in superior predictive performance. The GAM models were fit using the “mgcv” package version 1.8.31, with smoothing terms applied to the covariates using thin plate regression splines. E-net is a penalised regression model which incorporates both Lasso (L1) and Ridge (L2) regularisation as was fit using the “glmnet” package version 3.0.2, setting the α to 0.5 and the λ to the minimum value within one standard deviation of the λ of the best performing model. The estimates from the stacked ensemble model were then used as the explanatory covariates in the subsequent geostatistical model presented in Section 3.3.

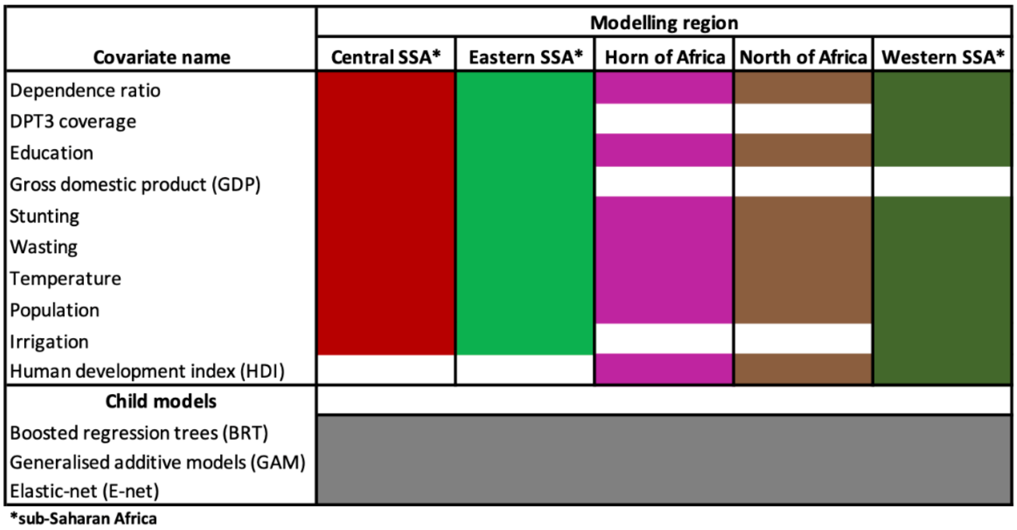

***Figure S5*: Model covariates selection for each modelling region.**

3.3 Geostatistical model

3.3.1 Model specification

To estimate the proportion of household overcrowding in each 5 x 5–km pixel in Africa we fit a spatially and temporally explicit binomial Bayesian hierarchical generalised linear model (GLM). We modelled the logit

probability of a household being overcrowded, p_i, using a GLM with the out-of-sample predictions from the stacked ensemble model, X_i which was used as the explanatory covariates with the coefficients, $\boldsymbol{\beta}$**,** constrained to sum up to one. A term for the residual spatial and temporal error was included, ε_{GP}; together with an independent nugget effect, ε_i; and a Gaussian country level random effect, ε_C, to allow for differences in household structure and composition, as well as housing policies' effects; and a Gaussian survey level random effect, $\epsilon_{S,}$ to account for biases and reporting inaccuracies.

The residual spatial and temporal error was modelled as a three-dimensional Gaussian process with a covariance matrix as the *Kronecker* product of spatial covariance ($\boldsymbol{K}_{\text{space}}$) and temporal covariance ($\boldsymbol{K}_{\text{time}}$). Spatial covariance was modelled as a stationary Matérn function[10] and temporal covariance as an autoregressive order 1 (AR1) function.

$$N_i^+ \sim Binomial(p_i, N_i)$$

$$logit(p_i) = \alpha + \boldsymbol{X}_i\boldsymbol{\beta} + \epsilon_C + \epsilon_S + \epsilon_i + \epsilon_{GP}$$

$$\epsilon \sim GP\left(0, \boldsymbol{K}_{space} \otimes \boldsymbol{K}_{time}\right)$$

$$\boldsymbol{K}_{space} = (2^{\nu-1}\Gamma(\nu))^{-1}(\kappa\boldsymbol{D})^{\nu}K_{\nu}(\kappa\boldsymbol{D})$$

$$\boldsymbol{K}_{time_{k,l}} = \rho^{\,|\mathrm{t_k}-\mathrm{t_l}|}$$

3.3.2 Spatial Mesh creation

A finite element spatial mesh is required to fit the stochastic partial differential equation (SPDE) approximation, used to model the spatial–temporal error. We used smoothed polygon boundaries for the modelled regions as templates for the meshes. We created inner triangles for the mesh with a maximum edge length set to 0·3 degrees and the outer buffer triangles maximum edge length set to 5 degrees. Figure S6 shows the mesh for the Central sub-Saharan Africa region. The spatial mesh was uniform for all modelled regions.

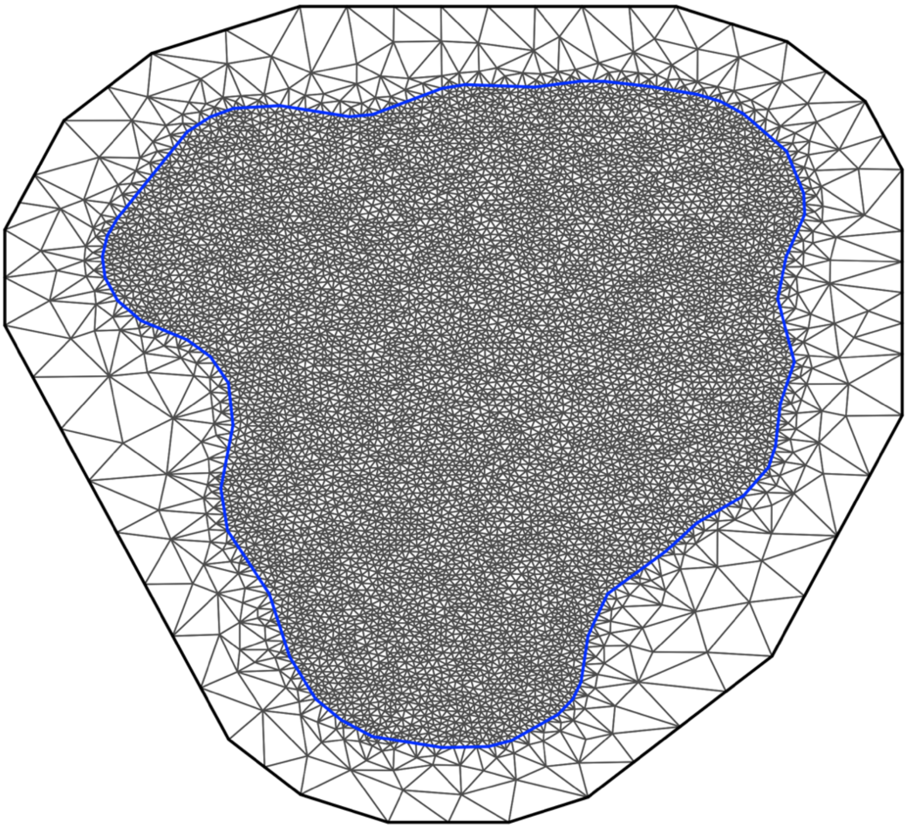

***Figure S6*: Finite element mesh for the Central sub-Saharan Africa region.**

3.4 Model fitting and validation

The models were fit using Integrated Nested Laplace Approximations (INLA)[11] in the R statistical software environment (R version 3.5.0)[12]. The spatial–temporal error was modelled using stochastic partial differential equation (SPDE) approximation with 1,000 draws of the model posterior. These were used to calculate the median, mean and 95% uncertainty intervals (UI) for the probability of household overcrowding for each 5 x 5–km pixel. The draw-level estimates were aggregated to the district, state and national levels, calculating the median, mean and 95% uncertainty intervals for each of these levels.

In order to inform how accurate the model predictions are where little or no data is present, we performed model predictive performance. Five-fold cross validation and computed out-of-sample predictive performance measures were performed (root mean square error (RMSE) and coefficient of determination (R^2)).

The models were validated following these steps:

a) Available surveys were assigned to one of five random folds;
b) Five datasets were created holding out one-fold of data from each dataset and fitting the model on each of these datasets using the final model specifications. Thus, creating an out-of-sample prediction for each data point;

c) The R^2 and RMSE of the input data points and the mean out-of-sample prediction were computed to show the predictive validity of the model. These metrics were computed for each level (district, state and national level metrics are presented in figures S11 - S12).

Household overcrowding model results

4.1 Model results

Household overcrowding proportions were estimated from 1,000 draws of the model posterior for each 5 x 5 km pixel in Africa for each year, from 2000 to 2018. The estimates were aggregated to the national (administrative level 0), state (administrative level 1) and district (administrative level 2) levels. Given policy decisions are often made at the district administrative level we present the estimates at this spatial resolution; this also provides identification of fine spatial trends without focusing on small populations. Figure S7 shows the district administrative level estimates of household overcrowding aggregated to five–year blocks throughout the study period (2000 - 2015).

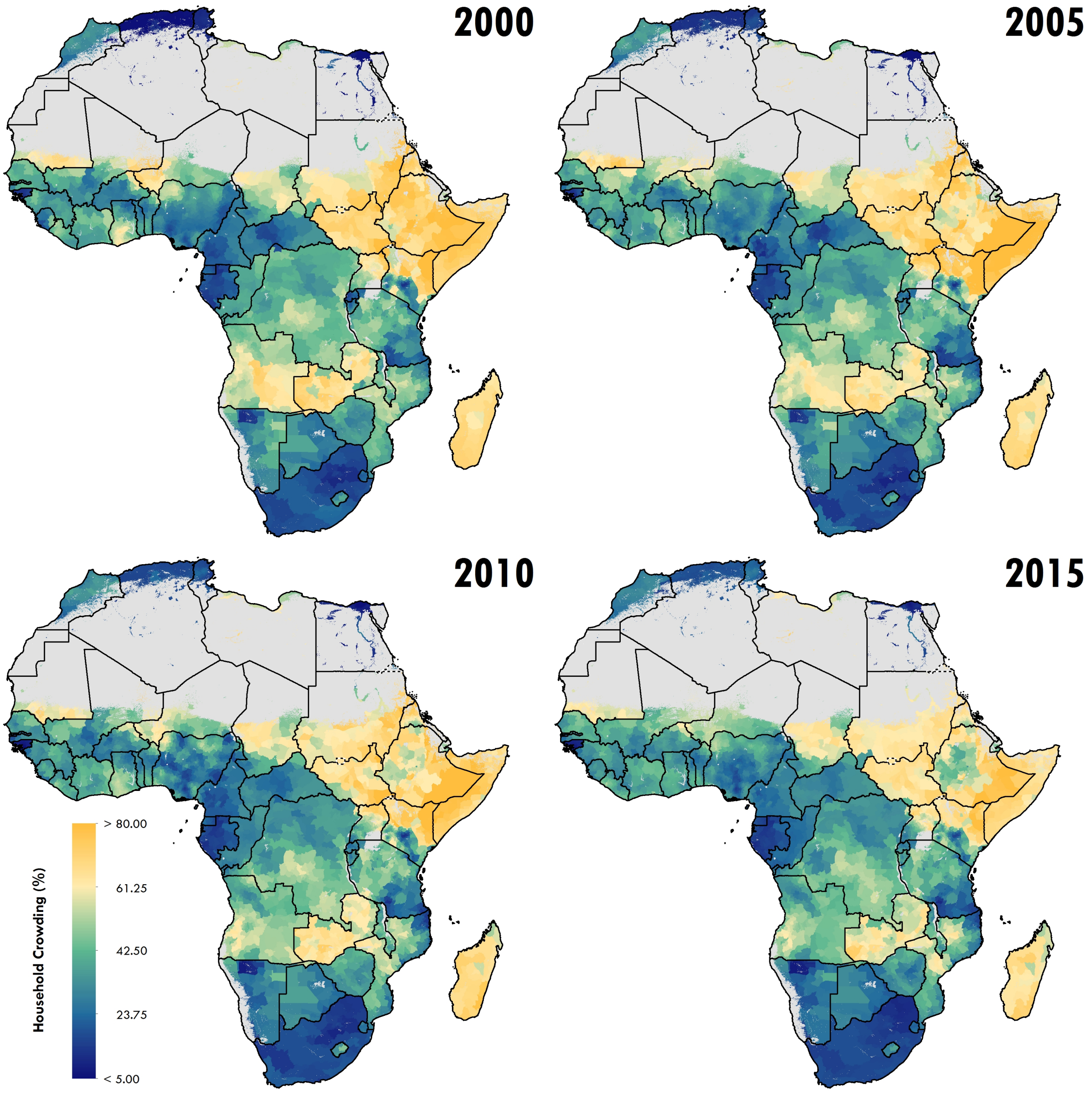

Figure S7: Five yearly estimates of the proportions of household overcrowding in Africa, at the administrative level 2 (district). Estimates for (a) 2000; (b) 2005; (c) 2010; (d) 2015. Pixels (1 x 1 km resolution) with populations of less than 10 are masked out in grey.

There has been very little change in household overcrowding over the study period across the Central-, Western-, Eastern & Southern- sSA. The Horn of Africa region, Zambia and Madagascar all recorded consistently high household overcrowding proportions. In contrast, a decrease in overcrowding proportions were estimated for

a number of countries: Ethiopia, down on average 17% (from 72% [95% UI: 67 - 77%] in 2000 to 55% [48 - 62%] in 2018); Angola decreased by 13% (from 56% [49 - 62%] to 43% [36 - 49%]) and Zambia decreased by 6% (from 63% [56 - 69%] to 57% [47 - 66%]); and (figure S8). Household overcrowding temporal trends showed an increase in the North of African countries: Algeria increased on average 14% (from 5% [95% UI: 2 - 11%] in 2000 to 19% [95% UI: 9 - 36%] in 2018) and Tunisia increased 11% (from 11% [95% UI: 3 - 25%] to 22% [95% UI: 9 - 42%]). Household overcrowding remained relatively low in South Africa throughout the study period, with an average of 15% [95% UI: 12 - 20%] in 2018.

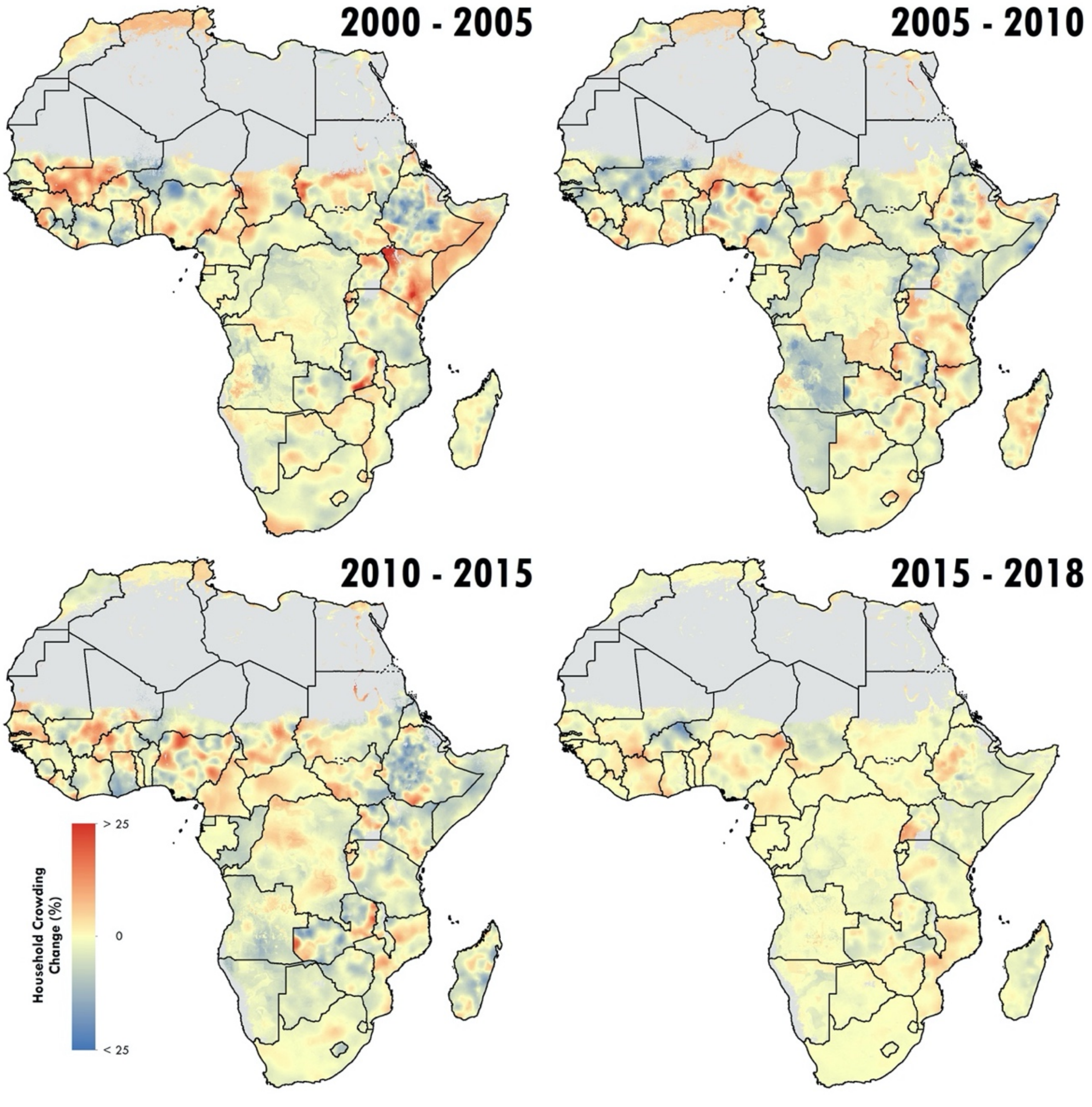

Figure S8: Five yearly estimates of change in the proportions of household overcrowding in Africa, at 5 x 5 km pixel level. Estimates for change (a) 2000 - 2005; (b) 2005 - 2010; (c) 2010 - 2015; (d) 2015 - 2018. Pixels (1 x 1 km resolution) with populations of less than 10 are masked out in grey.

We are able to use the model-based geospatial framework to model uncertainty quantification as shown in figure S9 where the lower, mean and upper uncertainty results are mapped (where (a) shows the 2·5 percentile, (b) the mean, and (c) the 97·5th percentile of the 1,000 draws of the model posterior). The 95% uncertainty range estimates are relatively small for Central-, Western-, Southern- sSA and North of Africa regions, indicating that the model is confident in predicting household overcrowding in these parts of Africa. However, the model estimates a relatively large range for the 95% uncertainty interval on Madagascar, Zambia, parts of Mozambique and the Horn of Africa regions as depicted by the density of colour in the upper 95% UI in figure S9.

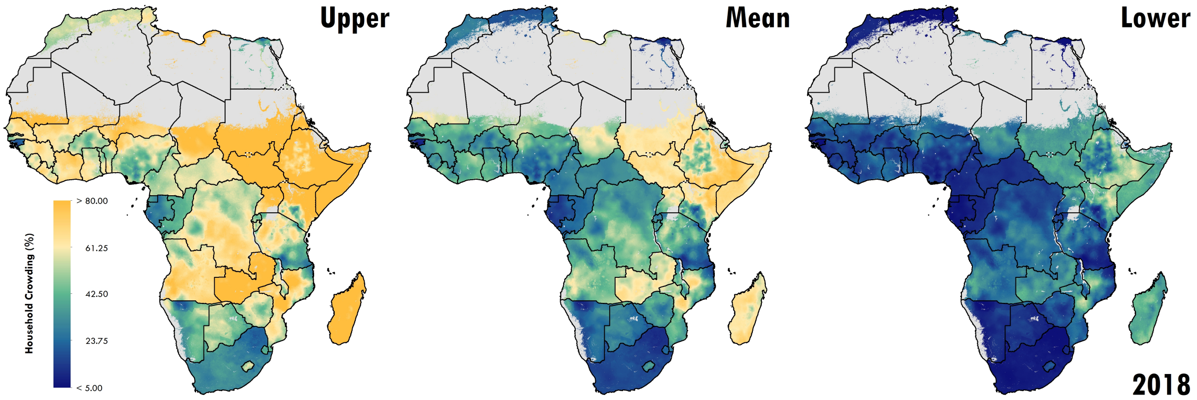

Figure S9: Estimates of the household overcrowding proportions in Africa for 2018 with the mean and upper and lower 95% uncertainty intervals. A) estimates of the upper 95% uncertainty value; b) mean estimates; c) estimates of the lower 95% uncertainty value. Pixels (1 x 1 km resolution) populations of less than 10 are masked out in grey.

Figure S10 compares the proportions of household overcrowding from low to high, versus relative uncertainty from the modelling, as measured by the ratio of 95% uncertainty interval to the mean in 2018. To quantify the level of household overcrowding we took the ratio of uncertainty intervals to the proportion of household crowding estimates in each district, and displayed this in relation to overcrowding proportion estimates. High relative uncertainty coupled with low household overcrowding proportions were observed in South Africa, Namibia, Botswana, Republic of Congo, Cameroon, CAR, Gabon, Equatorial Guinea, Guinea, Sierra Leone, Egypt, Tunisia, Algeria and Morocco. High overcrowding proportions and low relative uncertainty were observed in Madagascar, Zambia, Mozambique, Kenya, Somalia, Uganda, Ethiopia, South Sudan, Sudan, Southern parts of Chad and South Western parts of Angola. High household overcrowding proportions were observed with high uncertainty in most parts of Senegal, Mali, Niger, Liberia, Cote d'Ivoire, Ghana, Nigeria, Lesotho and DRC.

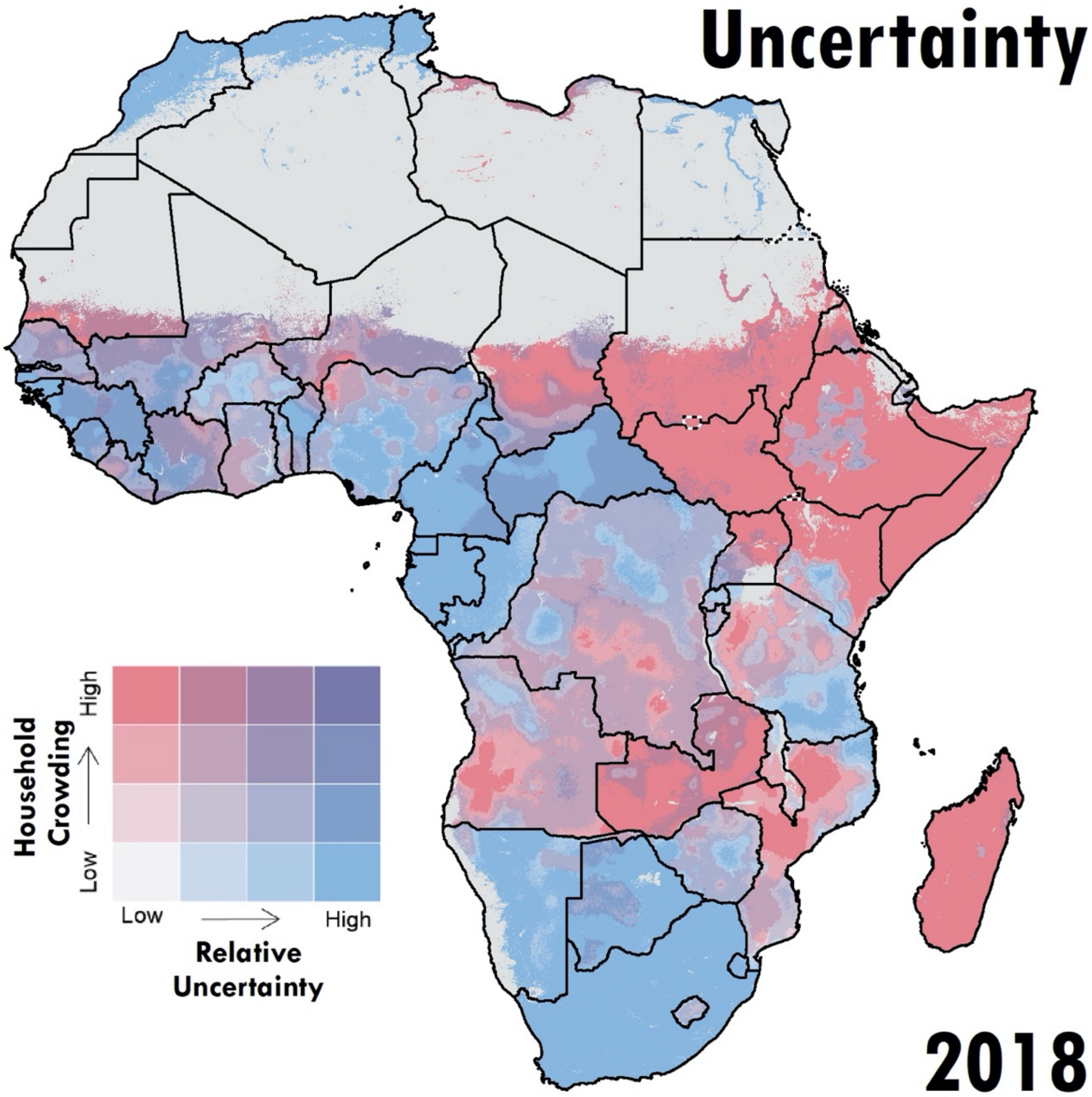

Figure S10: Overlapping population–weighted quartiles of household overcrowding and relative uncertainty in 2018. Relative uncertainty was computed as the ratio of the 95% uncertainty intervals and household overcrowding proportion for each pixel. The lowest quartile of household overcrowding is white, and the highest is dark pink. The lowest quartile for uncertainty is white and the highest is blue. These colours overlap such that areas coloured purple have both high overcrowding proportions and high relative uncertainty. Pixels (1 x 1 km resolution) with populations of less than 10 are masked out in grey.

4.2 Model validation

Out-of-sample model predictions were calculated as validation metrics, these were aggregated to the national level together with household overcrowding proportions from each survey from the input dataset. In order to understand the deviance of the model data from the input data we computed the root mean square error (RMSE) together with the R^2 to show the coefficient of determination between the data and model estimates. While the full model was correlated to the input data with an R^2 of 0·87 and an RMSE of 0·11; the predictive validity varied by region (table S3 and figures S12a - e) performing best in the Central sSA, North of Africa and Eastern & Southern sSA regions; and struggled for predictive validity in Western sSA, Eastern sSA and the Horn of Africa regions.

Table S3: In- and out-of-sample metrics for the five-fold cross validation models on household overcrowding.

Modelling region	In sample		Out of sample	
	RMSE	Corr* (R^2)	RMSE	Corr* (R^2)
**Overall**	0·08	0·95	0·11	0·87
**Eastern sub-Saharan Africa**	0·09	0·86	0·12	0·58
**Western sub-Saharan Africa**	0·08	0·74	0·12	0·27
**Central sub-Saharan Africa**	0·02	0·99	0·06	0·81
**North of Africa**	0·01	0·97	0·03	0·78
**Horn of Africa**	0·06	0·76	0·10	0·17

*Coefficient of determination.

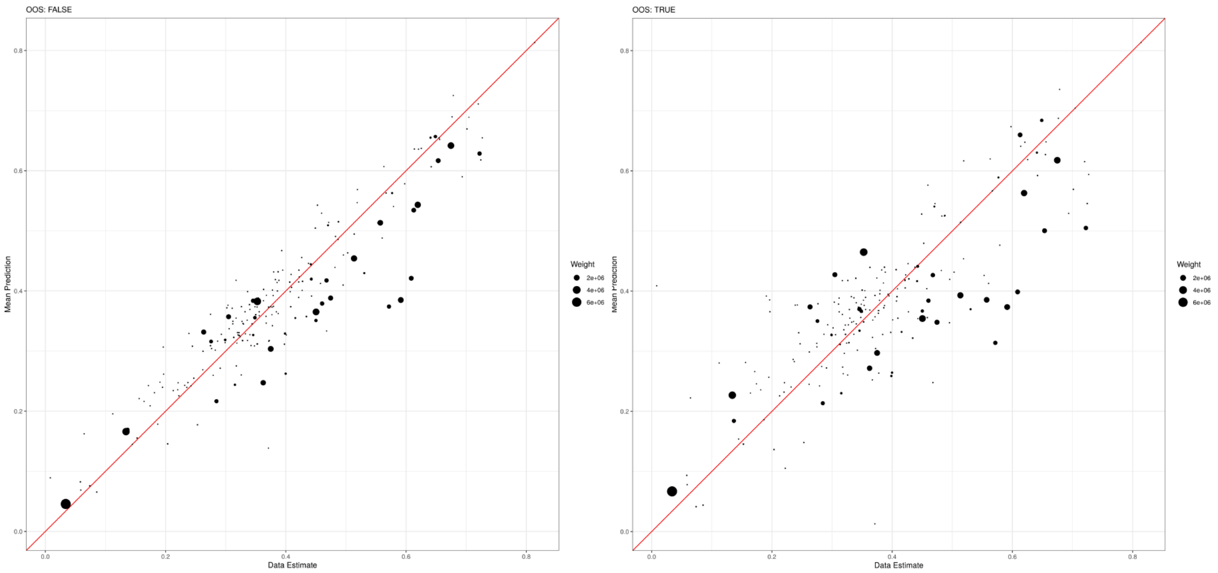

Figure S11: Validation plots for the model of household overcrowding proportions. a) Left scatter plot representing the mean proportion of household overcrowding for each survey, at the national level, against the in-sample predicted proportion of household overcrowding for that country–year; b) Right scatter plots of the mean proportion of household overcrowding for each survey, at the national level, against the out-of-sample predicted proportion of household overcrowding for that country based on five-fold cross validation.

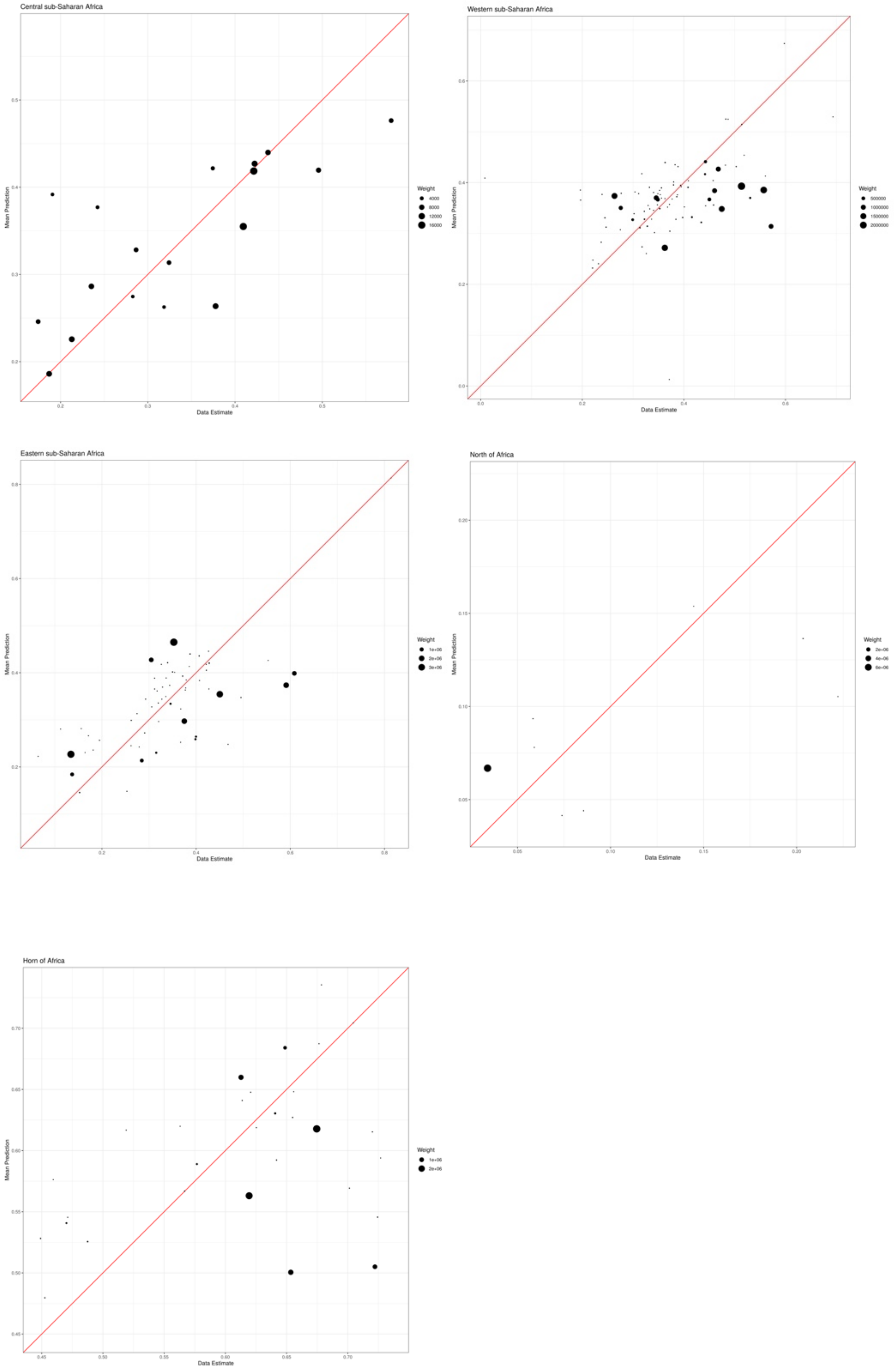

Figure S12: Validation plots for the model of household overcrowding for each modelling region. Each plot shows the mean household overcrowding proportion from each survey, at the national level, against the out-of-sample predicted household overcrowding proportion for that country–year

based on five-fold cross-validation. a) Central SSA; b) Western SSA; c) Eastern SSA; d) North of Africa; e) Horn of Africa.

Gather Checklist

This study was conducted in line with the Guidelines for Accurate and Transparent Health Estimates Reporting (GATHER). [13]

Table S4: GATHER Checklist. A checklist identifying each point of the Guidelines for Accurate and Transparent Health Estimates Reporting and whether they are covered in this study.

Item #	Checklist item	Reported on page #
**Objectives and funding**		
**1**	Define the indicator(s), populations (including age, sex, and geographic entities), and time period(s) for which estimates were made.	6, 9, S1
**2**	List the funding sources for the work.	3, 22
**Data Inputs**		
For all data inputs from multiple sources that are synthesized as part of the study:		
**3**	Describe how the data were identified and how the data were accessed.	7, 8, S3–S5
**4**	Specify the inclusion and exclusion criteria. Identify all ad-hoc exclusions.	8, S3
**5**	Provide information on all included data sources and their main characteristics. For each data source used, report reference information or contact name/institution, population represented, data collection method, year(s) of data collection, sex and age range, diagnostic criteria or measurement method, and sample size, as relevant.	7-9, S6–S67
**6**	Identify and describe any categories of input data that have potentially important biases (e.g., based on characteristics listed in item 5).	8
For data inputs that contribute to the analysis but were not synthesized as part of the study:		
**7**	Describe and give sources for any other data inputs.	7, 8, S66 – S67
For all data inputs:		
**8**	Provide all data inputs in a file format from which data can be efficiently extracted (e.g., a spreadsheet rather than a PDF), including all relevant meta-data listed in item 5. For any data inputs that cannot be shared because of ethical or legal reasons, such as third-party ownership, provide a contact name or the name of the institution that retains the right to the data.	S6 – S67
**Data analysis**		
**9**	Provide a conceptual overview of the data analysis method. A diagram may be helpful.	S68 – S69
**10**	Provide a detailed description of all steps of the analysis, including mathematical formulae. This description should cover, as relevant, data cleaning, data pre-processing, data adjustments and weighting of data sources, and mathematical or statistical model(s).	7 – 9, S65 – S70
**11**	Describe how candidate models were evaluated and how the final model(s) were selected.	8 – 9, S70
**12**	Provide the results of an evaluation of model performance, if done, as well as the results of any relevant sensitivity analysis.	S73 – S77
**13**	Describe methods for calculating uncertainty of the estimates. State which sources of uncertainty were, and were not, accounted for in the uncertainty analysis.	8 – 9, S70
**14**	State how analytic or statistical source code used to generate estimates can be accessed.	21
**Results and Discussion**		
**15**	Provide published estimates in a file format from which data can be efficiently extracted.	21
**16**	Report a quantitative measure of the uncertainty of the estimates (e.g., uncertainty intervals).	10 – 15, S70 – S74

**17**	Interpret results in light of existing evidence. If updating a previous set of estimates, describe the reasons for changes in estimates.	12 - 16
**18**	Discuss limitations of the estimates. Include a discussion of any modelling assumptions or data limitations that affect interpretation of the estimates.	15

References

1. Blake, K. S., Kellerson, R. L. & Simic, A. *Measuring overcrowding in housing*. (2007).
2. Gray, A. *Definitions of crowding and the effects of crowding on health: a literature review*. (2001).
3. UN-HABITAT. *Housing as a strategy for poverty reduction in Ghana United Nations human settlements programme*. (2010).
4. UK Government. Overcrowded households. (2020). Available at: https://www.ethnicity-facts-figures.service.gov.uk/housing/housing-conditions/overcrowded-households/latest. (Accessed: 10th July 2021)
5. Reynolds, L., Robinson, N. & Diaz, R. Crowded house Cramped living in England's housing From the Shelter policy library. (2004). Available at: www.shelter.org.uk. (Accessed: 10th July 2021)
6. Lloyd, C. T. High resolution global gridded data for use in population studies. *Int. Arch. Photogramm. Remote Sens. Spat. Inf. Sci. - ISPRS Arch.* **42**, 117–120 (2017).
7. Golding, N. *et al.* Mapping under-5 and neonatal mortality in Africa, 2000–15: a baseline analysis for the Sustainable Development Goals. *Lancet* **390**, 2171–2182 (2017).
8. World Health Organization. WHO Housing and Health Guidelines. (2018). Available at: https://www.ncbi.nlm.nih.gov/books/NBK535298/. (Accessed: 2nd August 2021)
9. Bhatt, S. *et al.* Improved prediction accuracy for disease risk mapping using Gaussian process stacked generalization. *J. R. Soc. Interface* **14**, (2017).
10. Matérn, B. *Spatial Variation*. (Springer, 1986).
11. Rue, H., Martino, S. & Chopin, N. Approximate Bayesian inference for latent Gaussian models using inte- grated nested Laplace approximations (with discussion). *J. R. Stat. Soc. Ser. B* **71**, 319–392 (2009).
12. R Core Team. R: A Language and Environment for Statistical Computing. (2017).
13. Stevens, G. A. *et al.* Guidelines for Accurate and Transparent Health Estimates Reporting: the GATHER statement. *Lancet* **388**, e19–e23 (2016).
